# The roles of RelA/(p)ppGpp in glucose-starvation induced adaptive response in the zoonotic *Streptococcus suis*

**DOI:** 10.1038/srep27169

**Published:** 2016-06-03

**Authors:** Tengfei Zhang, Jiawen Zhu, Shun Wei, Qingping Luo, Lu Li, Shengqing Li, Alexander Tucker, Huabin Shao, Rui Zhou

**Affiliations:** 1 State Key Laboratory of Agricultural Microbiology and Key Laboratory of Veterinary Diagnosis (Ministry of Agriculture), College of Veterinary Medicine, Huazhong Agricultural University, Wuhan 430070, China; 2 Hubei Key Laboratory of Animal Embryo and Molecular Breeding, Institute of Animal and Veterinary Science, Hubei Academy of Agricultural Sciences, Wuhan 430064, China; 3 Cooperative Innovation Center of Sustainable Pig Production, Wuhan 430070, China; 4 Department of Chemistry, College of Science, Huazhong Agricultural University, Wuhan 430070, China; 5 Department of Veterinary Medicine, University of Cambridge, Madingley Road, Cambridge, CB3 0ES, UK

## Abstract

The (p)ppGpp signal molecules play a central role in the stringent response (SR) to adapt to nutrient starvation in bacteria, yet the carbohydrate starvation induced adaptive response and the roles of SR in this response is not well characterized, especially in Gram-positives. Here, two (p)ppGpp synthetases RelA and RelQ are identified in *Streptococcus suis*, an important emerging zoonotic Gram-positive bacterium, while only RelA is functional under glucose starvation. To characterize the roles of RelA/(p)ppGpp in glucose starvation response in *S. suis*, the growth curves and transcriptional profiles were compared between the mutant strain Δ*relA* [a (p)ppGpp^0^ strain under glucose starvation] and its parental strain SC-19 [(p)ppGpp^+^]. The results showed great difference between SC-19 and Δ*relA* on adaptive responses when suffering glucose starvation, and demonstrated that RelA/(p)ppGpp plays important roles in adaptation to glucose starvation. Besides the classic SR including inhibition of growth and related macromolecular synthesis, the extended adaptive response also includes inhibited glycolysis, and carbon catabolite repression (CCR)-mediated carbohydrate-dependent metabolic switches. Collectively, the pheno- and genotypic characterization of the glucose starvation induced adaptive response in *S. suis* makes a great contribution to understanding better the mechanism of SR.

In mammalian hosts, bacterial pathogens suffer various challenges in environmental stress and nutrient insufficiency, for example, high temperature, acidic environment, ROS (Reactive oxygen species) stimulation, lack of amino acids, metal ions or carbon sources, and so on^1^,^2^,^3^,^4^,^5^,^6^. However, bacteria have evolved efficient stress response mechanisms to adapt to challenging environments. Among these responses, a special class of adaptive response induced by (p)ppGpp is called “stringent response (SR)”^7^. A wide array of physiological aspects, such as long-term persistence, virulence, biofilm formation and quorum sensing, have been reported to be affected by (p)ppGpp^8^,^9^,^10^,^11^.

The (p)ppGpp is synthesized by RelA/SpoT homologous proteins (RSH) through transferring a pyrophosphate moiety from ATP to GDP/GTP^12^,^13^,^14^. In *Escherichia coli* (*E. coli*) and some other Gram-negative bacteria, two RSH enzymes are involved in (p)ppGpp synthesis. The first enzyme RelA is recognized to respond to amino acid starvation in *E. coli*. The synthetic activity of RelA is activated by sensing ribosome idling reaction, and then (p)ppGpp accumulation leads to reprogramming transcription, decreasing synthesis of stable RNAs and ribosome proteins, and increasing amino acid biosynthesis^15^,^16^. Another homologous enzyme SpoT senses many other environmental stresses, such as starvation of carbon, iron, phosphate and fatty acids^7^,^17^,^18^. Different from *E. coli*, Gram-negative α-proteobacteria and Gram-positives contain a single RSH, such as Rel_*Bsu*_ in *Bacillus subtilis*^19^, Rel_*Mtb*_ in *Mycobacterium tuberculosis*^8^, and Rel_*Smu*_ in *Streptococcus mutans*^20^. Additionally, some small proteins with only (p)ppGpp synthetic activity are found in *Firmicutes*, such as *B. subtilis* and *S. mutans*^19^,^20^. Similar to RelA in *E. coli*, (p)ppGpp synthetic activity of RSH in Gram-positives can be also activated by interacting with idling ribosomes during amino acid starvation^12^. However, the mechanisms of SR induced by various stresses are different but not well characterized^7^. This includes the SR induced by carbon starvation.

Carbon resources are essential for all organisms. Carbon starvation causes a series of stress responses in bacteria, including the general stress response controlled primarily by carbon catabolite repression (CCR) and (p)ppGpp-mediated SR^21^,^22^,^23^. In *E. coli*, the genes regulated by regulators Crp and RpoS in CCR are RelA-dependent, implying that (p)ppGpp is at the apex of global regulation in carbon starvation^24^. However, the SR induced by carbon starvation is not yet fully characterized. The signaling pathway and global regulation during this process is largely unknown. About ten years ago, Battesti and Bouveret found that SpoT of *E. coli* can interact with the acyl carrier protein (ACP), the central cofactor of fatty acid synthesis. This interaction involves sensing the signals of fatty acid starvation, and triggering SpoT-dependent (p)ppGpp accumulation^25^. Given that carbon deprivation can lead to fatty acid starvation through shrinkage of the acetyl-CoA pool produced during glycolysis^26^, fatty acid metabolism could also be the relay for carbon starvation, another nutritional stress sensed by SpoT^7^,^25^. This opens the first window to understand how the RSH or SR is activated by carbon starvation. Three years later, the same research group demonstrated that the interaction between RSH and ACP occurs also in *Pseudomonas aeruginosa*, but not in the Gram-positive *B. subtilis* and *Streptococcus pneumoniae*^27^. These preliminary studies indicate that the regulatory mechanism of carbon starvation induced SR varies among different species of bacteria, while the analogous mechanisms in Gram-positives need to be characterized.

*Streptococcus suis* is a very important Gram-positive bacterium that causes deadly infections in pigs and humans. As an emerging zoonotic pathogen, *S. suis* serotype 2 has become the predominant causative agent of adult human meningitis in Vietnam and Hong Kong^28^. Two large outbreaks of human infections were reported in China in 1998 and 2005, resulting in 229 infections and 52 deaths^28^,^29^. As a bacterial pathogen, host adaptation is one of the most important steps for pathogenesis. The *relA* gene had been found to be up-regulated during iron starvation in our previous study^5^, suggesting that RelA may play an important role in the adaptive response to nutrient starvation in *S. suis*. Investigation of the mechanism of *S. suis* adaptation to environments will contribute to understanding the transmission and pathogenesis of this important zoonotic pathogen. Concerning that the regulatory behaviour and mechanism of (p)ppGpp synthetases on adaptive response to carbon starvation may be different in Gram positive bacteria from Gram negatives^27^, and remain unraveled, this study aims to characterize the (p)ppGpp synthetases and their regulation on adaptive response to carbon starvation in *S. suis*.

## Results

### Identification of (p)ppGpp synthetases in *S. suis*

**Figure 1:**
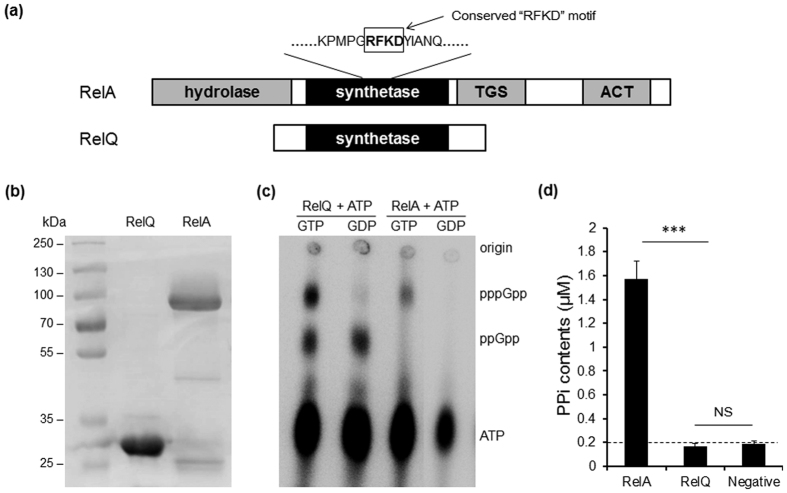
Expression and activity analysis of RelA and RelQ proteins of *S. suis*. (**a**) Domain structures of RelA and RelQ of *S. suis*. The RXKD motif was labeled in the box, TGS, conserved domain on RSH C-terminal domain for uncharged ACP binding in *E. coli*, ACT, a conserved regulatory domain on RSH C-terminal domain. (**b**) SDS-PAGE analysis of purified recombinant RelA and RelQ proteins. (**c**) Thin-layer chromatography (TLC) analysis of the (p)ppGpp synthesis activity of the recombinant RelA and RelQ. Purified RelA or RelQ protein is assayed for (p)ppGpp synthetase activity in the presence of [γ-P^32^]-ATP, 2 mM ATP, and either 1.3 mM GTP or GDP. Reaction mixtures were analyzed by TLC and autoradiography as described in Materials and Methods. (**d**) Hydrolase activity assays of recombinant RelA and RelQ by measuring the ppGpp hydrolysis product, PPi.

**Figure 2:**
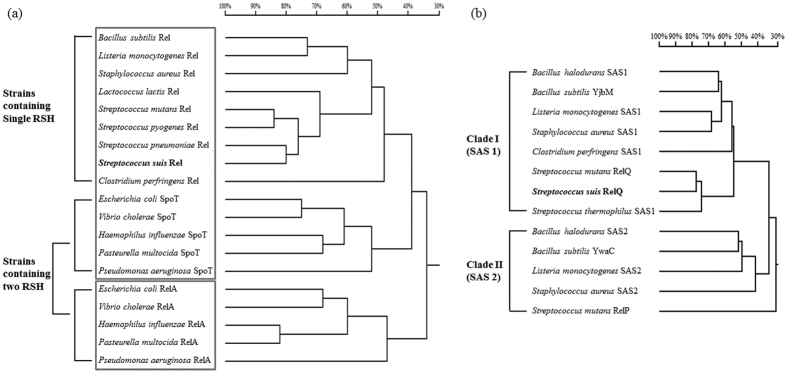
Phylogenetic trees for RelA-SpoT family proteins and SASs based on a multiple sequence alignment using DNAMAN. (**a**) Phylogenetic trees for RelA-SpoT family proteins; (**b**) Phylogenetic trees for SASs.

SR is an important adaptive response in bacteria, but until now, (p)ppGpp synthetase has not been characterized in *S. suis*. Searching the *S. suis* 05ZYH33 genome, we identified a RSH protein encoded by SSU05_2094 that consists of 733 amino acids. The N-terminal part is the catalytic domain for both hydrolysis and synthesis of (p)ppGpp, and the C-terminus is recognized as the regulatory domain^7^. This protein was named RelA. Further amino acids sequence analysis showed that RelA of *S. suis* contains a RXKD motif (Fig. 1a) and shows higher similarity with SpoT rather than RelA of *E. coli* and other strains which contain two RSH in the genome (Fig. 2a)^30^,^31^. Additionally, a paralogous protein HP1060 encoded by SSU05_1060, henceforth designated RelQ, was found. It only contains a putative (p)ppGpp synthetase domain, and lacks the hydrolase domain and regulatory domain (Fig. 1a), amino acids sequence analysis showed that the RelQ in *S. suis* had 78% identity with the RelQ in *S. mutans*. The phylogenetic tree revealed two clusters of the identified small alarmone synthetases (SASs) in *Firmicutes* and RelQ was clustered in the clade I group (Fig. 2b). To verify the (p)ppGpp synthetase activity of RelA and RelQ *in vitro*, soluble His-tagged recombinant RelA and RelQ were expressed in *E. coli*, purified (Fig. 1b) and then assayed for (p)ppGpp synthetase activity with [γ-^32^P]-ATP in the presence of 2 mM ATP and either 1.3 mM GTP or 1.3 mM GDP. The generated (p)ppGpp was detected by thin layer chromatography (TLC). The results showed that both ppGpp and pppGpp could be synthesized from GDP and GTP respectively by His-RelQ *in vitro*. Unexpectedly, ppGpp was detected when GTP was the substrate. It was a possible contaminant in the GTP that might arise from the intrinsic hydrolysis of GTP. In contrast, pppGpp could also be synthesized from GTP by His-RelA, but ppGpp was not detected when GDP was used as the substrate of His-RelA (Fig. 1c). The (p)ppGpp hydrolase activity of RelA and RelQ were further tested by detecting the hydrolytic product pyrophosphate (PP_i_) *in vitro* (Fig. 1d). There was no significant difference on the levels of PPi in the RelQ reaction (0.17 ± 0.02 μM) and negative control (0.19 ± 0.019 μM, no protein was added) (*p* > 0.05). We inferred that the small quantity of PPi came from the intrinsic hydrolysis of (p)ppGpp during storage or experiments. In contrast, approximately 1.57 μM PP_i_ was detected in the RelA-catalyzed hydrolysis reaction. These results demonstrated that RelA is a bifunctional enzyme, which could synthesize and hydrolyze (p)ppGpp, whereas RelQ is a monofunctional (p)ppGpp synthetase in *S. suis*.

### (p)ppGpp accumulation by *S. suis* strains during glucose starvation

**Figure 3:**
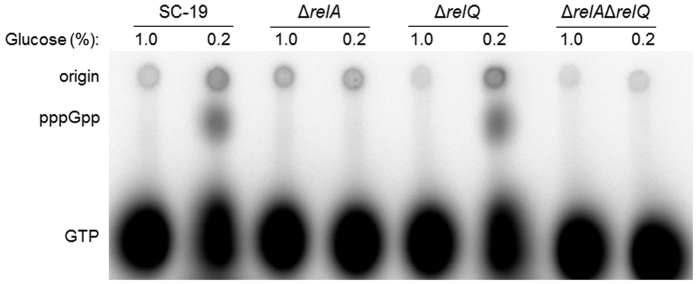
The (p)ppGpp production by *S. suis* SC-19 and its mutant strains during glucose starvation. The strains SC-19, Δ*relA*, Δ*relQ* and Δ*relA*Δ*relQ* were grown in the MOPS-CDM containing 150 μCi/ml H_3_[^32^P]O_4_, and 1.0% or 0.2% glucose at 37 °C for 30 min. The formic acid extracts of the cells were subjected to TLC analysis as described in Materials and Methods.

**Figure 4:**
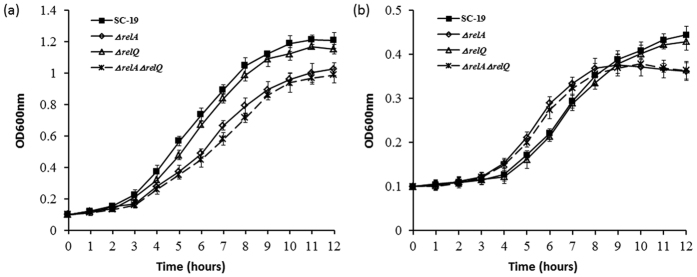
Growth curves of *S. suis*. The SC-19 and its mutant strains Δ*relA*, Δ*relQ* and Δ*relA*Δ*relQ* were grown in the CDM containing 1.0% glucose (**a**) or 0.2% glucose (**b**), respectively. Each curve shown is representative of a typical experiment that was performed three times.

To further verify the function of RelA and RelQ during glucose starvation, the mutant strains Δ*relA*, Δ*relQ* and Δ*relA*Δ*relQ* were constructed using homologous recombination method and verified by PCR, RT-PCR and Southern blot. The (p)ppGpp accumulation in SC-19 and the three mutant strains during glucose starvation was studied by TLC analysis. When cells were cultured in the complete CDM, no (p)ppGpp was detected in all tested strains. In contrast, when cultured in the glucose-deficient CDM, (p)ppGpp synthesis could be detected in the *relA*-positive strains (SC-19 and Δ*relQ*), but not *relA*-deletion strains (Δ*relA* and Δ*relA*Δ*relQ*) (Fig. 3). We further detected the ppGpp contents by anion exchange high performance liquid chromatography (HPLC) at the time point of 5 h, the sample collection point for microarray analysis (Fig. 4). When cultured in CDM containing 0.2% glucose, 119 ± 15 and 105 ± 19 pmoles/ml ppGpp were detected in SC-19 and Δ*relQ* respectively, while no ppGpp was detected in *relA*-deletion strains. No ppGpp was detected in all the strains in CDM containing 1% glucose. These results indicated that glucose starvation can cause (p)ppGpp accumulation in *S. suis*, wherein RelA is the sole functional (p)ppGpp synthetase during glucose starvation. Whereas, although RT-PCR test have proved that *relQ* gene was expressed under glucose starvation, RelQ protein did not synthesize (p)ppGpp in Δ*relA* under this condition.

### Growth of *S. suis* strains during glucose starvation

To investigate growth performance of the strains during carbon starvation, the wild type strain SC-19 and 3 mutant strains were grown in the complete CDM (containing 1% glucose) and glucose-deficient CDM (containing 0.2% glucose), respectively. In the complete CDM, RelQ inactivation did not affect the growth of *S. suis*, while the growth of RelA inactivated strains (Δ*relA* and Δ*relA*Δ*relQ*) slowed down when compared with SC-19 and Δ*relQ* (Fig. 4a). In the glucose-deficient CDM, the growth rate of all strains was significantly decreased when compared with that in the complete CDM; RelA inactivated strains (Δ*relA* and Δ*relA*Δ*relQ*) showed a higher growth rate than SC-19 and Δ*relQ* at the beginning of starvation, and then more quickly turned into stationary phase; Δ*relQ* displayed the similar growth performance as SC-19 (Fig. 4b). These results indicated that it is RelA but not RelQ contributing to the growth regulation of *S. suis* during glucose starvation.

### Overview of microarray data

**Figure 5:**
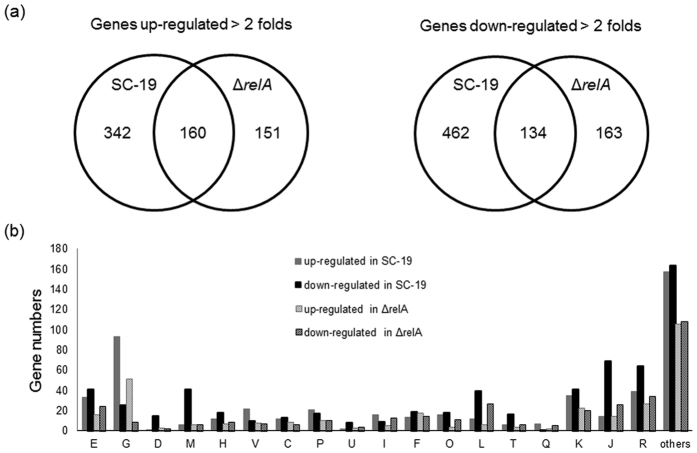
Summary of the differential expressed (DE) genes in *S. suis* SC-19 and Δ*relA* response to glucose starvation (growth in 0.2% glucose vs 1% glucose). (**a**) Venn diagrams show the up- (*left*) and down-regulated (*right*) genes. (**b**) The DE genes are classified into 19 functional categories. E, Amino acid transport and metabolism; G, Carbohydrate transport and metabolism; D, Cell cycle control, mitosis and meiosis; M, Cell wall/membrane biogenesis; H, Coenzyme transport and metabolism; V, Defense mechanisms; C, Energy production and conversion; P, Inorganic ion transport and metabolism; U, Intracellular trafficking and secretion; I, Lipid transport and metabolism; F, Nucleotide transport and metabolism; O, Posttranslational modification, protein turnover, chaperones; L, Replication, recombination and repair; T, Signal transduction mechanisms; Q, Secondary metabolites biosynthesis, transport and catabolism; K, Transcription; J, Translation; R, General function prediction only.

Growth curves and (p)ppGpp accumulation assays showed that RelA inactivation could influence *S. suis* growth and led to incapacity of (p)ppGpp synthesis during glucose starvation (Figs 3 and 4). To identify the roles of RelA/(p)ppGpp in global gene regulation in *S. suis*, we compared the transcriptional profiles of SC-19 [a (p)ppGpp^+^ strain] and Δ*relA* [a (p)ppGpp^0^ strain during glucose starvation] in both glucose-abundant and -deficient CDM in early exponential phase by microarray analysis. We considered genes to be significantly induced or repressed if the absolute value of the expression ratio was > two-fold. qRT-PCR validation displayed the same trends observed in the microarrays (see Supplementary Tables S1 and S2). The results showed that 502 genes were up-regulated and 596 genes were down-regulated in SC-19, while 311 genes were up-regulated and 297 genes were down-regulated in Δ*relA* during glucose starvation (see Fig. 5a and Supplementary Tables S3 and S4). More genes were found to be differentially expressed (DE) in SC-19 compared to Δ*relA* during glucose starvation, suggesting a globally regulatory role of RelA/(p)ppGpp in *S. suis* under this condition. According to COG classification, the DE genes were classified into 19 categories (Fig. 5b). Among them were large numbers of genes involved in carbohydrate transport and metabolism, transcription and translation. Of note, plenty of genes in the glycolysis and extended metabolism pathways were differentially regulated between SC-19 and Δ*relA*. In addition, many genes associated with cell cycle and cell wall/membrane biogenesis were down-regulated in SC-19, but not in Δ*relA*. The details of these regulated genes are described below.

### Typical (p)ppGpp-dependent SR

**Figure 6:**
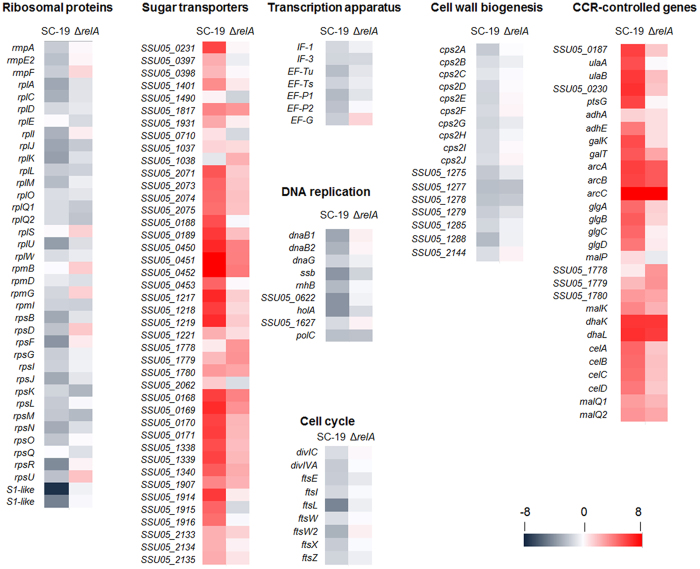
Heat maps of some important DE genes in the *S. suis* response to glucose starvation. Heat maps of log 2 expression ratios for the SC-19 and Δ*relA* for the ribosomal protein genes, sugar transporter, CCR controlled genes and genes related to transcription apparatus, DNA replication, cell cycle and cell wall biogenesis are shown.

An important feature of classical SR is inhibition of biomacromolecule synthesis^15^. In this study, protein synthesis was inhibited by glucose starvation in SC-19. Compared to 26 DE genes in Δ*relA*, more genes (69 genes) associated with protein translation were down-regulated in SC-19 (Fig. 6). Hereinto, 34 ribosomal protein genes were down-regulated in SC-19. The genes encoding transcription apparatus were also down-regulated by glucose starvation in SC-19, such as translation initiation factors IF-1 (SSU05_0092) and IF-3 (SSU05_1270), translation elongation factors EF-Tu (SSU05_0530), EF-Ts (SSU05_1979), EF-G (SSU05_0922) and EF-P (SSU05_1823, SSU05_1824). This is compatible with the proposed mechanism of direct down-regulation of rRNA synthesis during SR^7^. Different from in SC-19, the expression changes of the protein synthesis related genes in Δ*relA* were not regular during glucose starvation. In Δ*relA*, 11 genes encoding ribosomal proteins were down-regulated, while another 6 were up-regulated. At the same time, translation initiation factors and translation elongation factors was not obviously repressed except IF-3. These results suggested that RelA/(p)ppGpp inhibited the protein synthesis by down-regulating the expression of ribosomal proteins and translation factors during glucose starvation.

Besides protein synthesis, DNA replication was also inhibited in SC-19 during glucose starvation. These down-regulated genes including: *dnaB* (SSU05_2158, SSU05_2159) encoding DNA helicase, *ssb* (SSU05_1833) encoding single-stranded DNA binding protein, *dnaG* (SSU05_1429) encoding primase, *rnhB* (SSU05_0996) encoding ribonuclease HII and four subunits of DNA polymerase III (SSU05_0662, SSU05_1540, SSU05_1627 and SSU05_1954). In contrast, only two subunits of DNA polymerase III (SSU05_1540, SSU05_1954) and *ssb*, which were related to DNA replication, were regulated in Δ*relA*. These results suggested that RelA/(p)ppGpp inhibited the DNA replication by down-regulating the relative enzymes during glucose starvation.

Meanwhile, the expression of cell division and growth related genes were repressed in SC-19 during glucose starvation as well. Fourteen genes encoding essential proteins for cell division were down-regulated in SC-19, including *ftsE* (SSU05_1411), *ftsI* (SSU05_1354), *ftsL* (SSU05_1743), *ftsW* (SSU05_0526), *ftsX* (SSU05_1410), *ftsZ* (SSU05_0481), *divIVA* (SSU05_0487) and *divIC* (SSU05_0010). In contrast, only one gene encoding actin-like ATPase (SSU05_0479) involved in cell division was down-regulated in Δ*relA*. These results suggested that RelA/(p)ppGpp inhibited the expression of cell division relative proteins during glucose starvation.

Finally, the expression of cell wall/membrane biogenesis genes was inhibited by glucose starvation in SC-19. These include the CPS biosynthesis gene cluster (*cps2ABCDEFGHIJ*, SSU05_0564-0573), genes encoding glycosyltransferases for cell wall biosynthesis (SSU05_1275, SSU05_1277 and SSU05_2144) and polysaccharide biosynthesis proteins (SSU05_1285, SSU05_1288). Comparing with SC-19, the expression of these genes was not obviously changed in Δ*relA*. These results suggested that *cps* gene cluster was also inhibited by RelA/(p)ppGpp during glucose starvation.

### Regulation on carbohydrate transport

The phosphoenolpyruvate-dependent phosphotransferase system (PTS) and ATP-binding cassette (ABC) transporters are two major mechanisms for carbohydrates uptake in bacteria^22^. As glucose is the first-line carbon source for most bacteria, glucose starvation caused obvious changes on expression of the genes involved in carbohydrate transport. In SC-19, the expression of 27 PTS components and 4 ABC-type sugar transporters were up-regulated. In contrast, only 13 PTS components were up-regulated while 2 PTS components down-regulated in Δ*relA*. Besides *lacEF* (SSU05_1037-1038), transcription of 11 of the 13 up-regulated PTS components were also induced in SC-19. Two ABC sugar transporters which were highly expressed in SC-19 were also up-regulated in Δ*relA*. Although many genes were up-regulated in both strains, the fold changes of most regulated sugar transport systems in SC-19 were greater than those in Δ*relA* (Fig. 6). These results suggested that RelA/(p)ppGpp contributed to the expression of carbohydrate transporters during glucose starvation.

### Regulation on glycolysis

**Figure 7:**
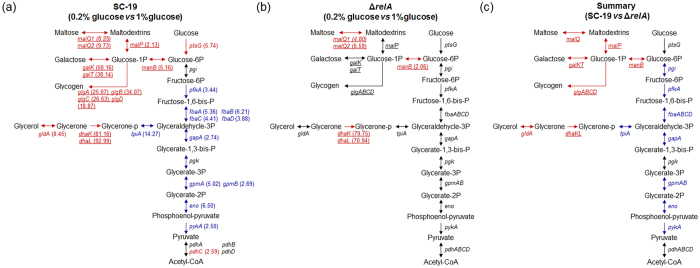
Regulation of glucose starvation on glycolysis and carbohydrate utilization in *S. suis* SC-19 (**a**), Δ*relA* (**b**) and the summary of SC-19 vs Δ*relA* (**c**). Transcriptome data is overlaid on the selected metabolic pathways. Genes up-regulated > two-fold are shown in red, while genes down-regulated > two-fold are shown in blue. Genes whose expression is not changed up to two-fold are shown in black. The *cre*-box containing genes are marked by underline.

The roles of RelA/(p)ppGpp in glycolysis, and its interrelated pathways in *S. suis*, were interpreted using the microarray data in a central metabolic context. Gene expression ratios were overlaid onto central carbon metabolic maps (Fig. 7). The expression levels of genes between SC-19 and Δ*relA* in glycolysis showed obvious difference. In glycolytic pathway, *fbaABCD* (SSU05_0336-0339), tpiA (SSU05_0531), *gapA* (SSU05_0155), *gpmAB* (SSU05_1638, SSU05_0520) and *eno* (SSU05_1503) encoding five reversible enzymes, which could convert fructose-1,6-bis-P to phosphoenol-pyruvate, were more than two-fold down-regulated in SC-19 during glucose starvation. Because gluconeogenesis is incomplete in *S. suis*, the main metabolic direction is from glucose to pyruvate^32^. More to the point, *pfkA* (SSU05_0543) and *pykA* (SSU05_0544), encoding 6-phosphofructokinase and pyruvate kinase respectively, which catalyzed two irreversible steps in glycolysis, were down-regulated in SC-19. In contrast, the expression level of genes in glycolysis did not change up to two fold in Δ*relA* during glucose starvation. These results implied that glycolysis was repressed in *S. suis* by RelA/(p)ppGpp during glucose starvation (Fig. 7c).

### RelA/(p)ppGpp influences carbon catabolite repression

Carbon catabolite repression (CCR) is the most important regulation mechanism in bacteria when suffering carbon starvation^33^. Therein, catabolite control protein A (CcpA) and transcriptional anti-terminator containing PTS-regulatory domain (PRD) are the key regulators^22^,^34^,^35^. In order to find the connection between SR and CCR during glucose starvation, we focused on the genes regulated by both RelA/(p)ppGpp and CCR. The *cre*-box, the target of the negative regulator CcpA, was scanned in *S. suis* genome using Virtual Footprint^33^. 48 and 26 DE genes/operons were found to contain a *cre*-box in their promoter regions in SC-19 and in Δ*relA*, respectively. Most of these genes were related to utilization of sugar resources including galactose, glycogen, maltose and glycerol, and some specific amino acids (Fig. 7). Some *cre*-dependent operons were only up-regulated in SC-19. For example, the operon *galKT* (SSU05_0360, SSU05_0361) encoding the enzymes for conversion of galactose to Glc-1-p, was more than 38-fold overexpressed in SC-19, suggesting that use of galactose during glucose starvation was very important in *S. suis*^36^. The *glgABCD* operon (SSU05_1013–1016) encoding the enzymes for glycogen biosynthesis^37^ was approximately 20-fold up-regulated in SC-19 during glucose starvation. Another set of regulated genes were up-regulated both in SC-19 and Δ*relA*, but induction was maximized in SC-19. For example, *malQ1* and *malQ2* (SSU05_2131 and SSU05_2132) encoding the 4-α-glucanotransferase, were up-regulated in both SC-19 and Δ*relA*, and *malP* (SSU05_1444) encoding maltodextrinphosphorylase, was up-regulated only in SC-19. These enzymes can convert maltose to Glc-1-p, and their high-level expression may assist *S. suis* in using maltose^38^. The *dhaKL* (SSU05_1957, SSU05_1958), which encode the dihydroxyacetone kinases involved in glycerol utilization, were more than 80-fold and 70-fold induced in SC-19 and Δ*relA* respectively, suggesting glycerol was also an important carbon source for *S. suis* during glucose starvation. The genes encoding enzymes associated with alcohol utilization were also up-regulated, including *adhA* (SSU05_0279) and *adhE* (SSU05_0280). The *arcABC* operon (SSU05_0624, SSU05_0626, SSU05_0627) encoding the arginine deiminase system (ADS) was significantly up-regulated in both SC-19 and Δ*relA*, and the fold change of this operon in SC-19 was as twice as that in Δ*relA*. ADS catalyzes the fermentative catabolism of arginine and generates an ATP per one arginine consumed^39^. ADS may be another important way for energy supply in *S. suis* during glucose starvation. These genes/operons were typically *cre*-dependent and regulated by CcpA. However, when analyzing the expression levels of these genes/operons, we found that RelA/(p)ppGpp was required for induction or maximal induction of them during glucose starvation (Fig. 6).

Some PTS operons, containing a transcriptional anti-terminator with a PTS-regulatory domain (PRD), are regulated by phosphorylating PRDs^22^. We found that some PRD-containing operons were significantly differently expressed between SC-19 and Δ*relA* during glucose starvation, such as *ulaAB* and *celABC* which govern uptake of galactitol and cellobiose. The *ulaAB* operon (SSU05_0188 and SSU05_0189) carrying a PRD-containing transcriptional anti-terminator (SSU05_0187) was more than 46 fold up-regulated in SC-19, however, only less than 4 fold up-regulated in Δ*relA*. Another transcriptional anti-terminator (SSU05_2076) containing PTS *celABC* (SSU05_2073–2075) was approximately 20 fold up-regulated in SC-19. These results showed that induction or maximal induction of these PRD-containing operons also required RelA/(p)ppGpp (Fig. 6).

## Discussion

Environmental adaptation is an important issue for bacterial survival, growth and pathogenesis, and SR is one of the most important mechanisms for environmental adaptation^8^,^40^. The signal molecules (p)ppGpp are the key player in SR induced by nutrient starvation^14^,^16^,^41^. In this study, two (p)ppGpp synthetases are identified in the emerging zoonotic pathogen *S. suis*. One is RelA containing all the four RSH domains, and another is RelQ which only contains a (p)ppGpp synthetase domain (Fig. 1a). The *relA* gene was found to be up-regulated in *S. suis* during iron starvation in our previous study^5^, suggesting that *relA* may play a role in environmental adaptation and pathogenesis of *S. suis*. The synthetase and hydrolase activities of RelA were shown by the *in vitro* assays (Fig. 1), although RelA did not show the ability to synthesize ppGpp (Fig. 1c). Sajish *et al.* has reported that, bifunctional (synthesis and hydrolysis) RSH like SpoT in *E. coli*, contains a RXKD motif and prefer to utilize GTP, while monofunctional (synthesis) RSH like RelA in *E. coli* contains an EXDD motif and prefer to utilize GDP^30^,^31^. Sequence analysis showed that RelA in *S. suis* is closer to SpoT but not to RelA in *E. coli*, and it has the typical characteristic in bifunctional RSH that contains a RXKD motif. That in *S. suis* the preference of RelA on GTP rather than GDP may be the reason why no ppGpp was detected in present of GDP. At the same time, we report for the first time RelQ and its (p)ppGpp synthetic activity in *S. suis in vitro* (Fig. 1c). Previous studies have identified several SASs which had the (p)ppGpp synthetic capacity in members of class *Firmicutes*, such as YjbM and YwaC in *B. subtilis*, and RelP and RelQ in *Streptococcus mutans*^19^,^20^. The RelQ identified in *S. suis* had 78% identity of amino acid sequence with the RelQ in *S. mutans*, which could synthesize (p)ppGpp during amino acid starvation in *S. mutans*^20^, we infer that RelQ in *S. suis* is also functional during amino acid starvation. Unexpectedly in Fig. 1c, there was an excrescent point ppGpp when GTP was used as the substrate of RelQ. Recently, Anthony *et al.* reported that RelQ_EF_ synthesizes ppGpp more efficiently than pppGpp^42^, that may be the reason why ppGpp was detected largely in our test when possible GDP contamination existed in GTP. In *Bacillus subtilis*, initiative suppressor mutations in SASs could partially relieve the growth defect of *relA* mutant^43^. However, no initiative mutation was found in the *relQ* gene of *S. suis relA* mutant.

Carbohydrates are important nutrients for most bacteria, and carbon starvation can induce SR in many species of bacteria, whereas its mechanisms are largely unclear, especially in Gram-positives^24^,^27^. To disclose the functional roles of the RSH proteins RelA and RelQ in regulation of SR in *S. suis*, we constructed three isogenic mutant strains Δ*relA*, Δ*relQ* and Δ*relA*Δ*relQ*. Their (p)ppGpp accumulation during glucose starvation was detected by TLC and HPLC analysis. It is interesting that although *relQ* was expressed on transcriptional level in both SC-19 and Δ*relA* during glucose starvation, (p)ppGpp can only be detected in the *relA*-positive strains (SC-19 and Δ*relQ*) but not in *relA*-deleted strains (Δ*relA* and Δ*relA*Δ*relQ*) (Fig. 3), demonstrating that RelA was the sole functional (p)ppGpp synthetase in *S. suis* during glucose starvation (i.e. RelQ is not functional under this condition). Thus, under glucose starvation, Δ*relA* can be considered as a (p)ppGpp^0^ strain of *S. suis* and was used to study the regulatory functions of (p)ppGpp.

To characterize the roles of RelA/(p)ppGpp in glucose-starvation induced adaptive response in *S. suis*, we compared and analyzed the glucose starvation responses of SC-19 and Δ*relA* on growth curves and transcriptional profiles in this study. Comparing with SC-19, Δ*relA* had a little lower growth rate when grown in the CDM containing 1% glucose (Fig. 4a). In contrast in the CDM containing 0.2% glucose, the growth of SC-19 lagged Δ*relA* at the beginning of glucose starvation, but then Δ*relA* declined more quickly (Fig. 4b). The different growth phenotypes between SC-19 and Δ*relA* suggested that RelA/(p)ppGpp regulates the growth of *S. suis* during glucose starvation. These phenotypes were also reflected on the transcriptional levels of related genes from the results of microarray, including the typical SR on macromolecular synthesis and a series of special regulation on carbon metabolism. DNA replication, protein translation, cell division and cell wall/membrane biogenesis were significantly inhibited during glucose starvation in SC-19 (Fig. 6). These changes on macromolecular synthesis are the typical RelA/(p)ppGpp-mediated SR, which could be induced by amino acid starvation in many bacteria^16^,^44^,^45^. At the same time, glucose starvation induces a special regulation pattern on carbon metabolism in SC-19: all the glycolysis-related genes were down-regulated, while the CCR controlled genes were significantly activated by glucose starvation in SC-19 (Fig. 7a), suggesting that SC-19 has adjusted to reduce consumption of glucose and tries its best to uptake and utilize other carbon resources, such as galactose, maltose, glycerol, and even amino acids like arginine (Fig. 6). In contrast, glucose starvation does not regulate the glycolysis-related genes in Δ*relA* (Fig. 7b). It is well known that RelA/(p)ppGpp-mediated SR directly inhibit the macromolecular synthesis^15^, in this study we could conclude that, RelA/(p)ppGpp also plays important roles on the regulation of the glycolysis-related genes during glucose starvation adaptive response. Taken together, *S. suis* inhibits its macromolecular synthesis, cell cycle and adjusts its carbohydrate metabolisms to adapt to glucose starvation for long-term survival.

It is well known that CCR is a key player on regulation of the uptake and utilization of carbon resources^32^. In the present study, different transcriptional patterns of the CCR regulons were observed between SC-19 and Δ*relA* under glucose starvation (Fig. 7), indicating an important role of RelA/(p)ppGpp in this process. For example, some CCR regulons such as *glgABCD*, *galKT* and *malP* were only up-regulated in SC-19 but not in Δ*relA*, while other CCR regulons such as *malQ*, *dhaKL* and *manB* were induced at higher levels in SC-19 than in Δ*relA*. This may be due to the different expressional levels of genes in glycolysis between SC-19 and Δ*relA*. In glucose metabolism, the intermediates and their derivatives in glycolysis act as the indicators for CCR control, such as fructose-1,6-bisphosphate, and glucose-6-phosphate^22^. During glucose starvation, the inhibited glycolysis in SC-19 leads to decreased concentration of the indicators, and consequently results in significant derepression and up-regulation of the CCR regulons. In contrast, the transcriptional levels of the genes in glycolytic pathway do not changed in Δ*relA* [(p)ppGpp^0^]. These findings indicate that an intersectional link should exist between the RelA/(p)ppGpp and CCR systems, at least in regulation of the carbon starvation induced SR.

Early studies have demonstrated that fatty acid (carbon) starvation is sensed by ACP/SpoT interaction in *E. coli*, and the C-terminal TGS domain of SpoT is essential for this interaction^25^,^27^. However, our Bacterial Two-Hybrid analysis demonstrated that neither RelA nor RelQ can interact with ACP in *S. suis* (see Supplementary Fig. S1). Therefore, the signaling pathway of carbon starvation-induced SR in *S. suis* is different from that in *E. coli*. We infer that there might be other protein(s) other than ACP functioning as RSH-interacting partner(s) to transmit the carbon starvation signal in *S. suis*. Given that (p)ppGpp could not be synthesized in Δ*relA* by RelQ during glucose starvation, we speculate that it might be due to absence of the carbon-starvation-sensing domain in RelQ. We currently are trying to identify the potential RelA-interacting partner(s) that can sense carbon starvation and induce stringent response in the zoonotic pathogen *S. suis*.

In conclusion, two (p)ppGpp synthetases RelA and RelQ are identified in *S. suis*, while only RelA is functional during glucose starvation. The RelA/(p)ppGpp-mediated stringent response plays important roles in adaptation to glucose starvation. Besides the classical SR including inhibition of growth and related macromolecular synthesis, the glucose-starvation induced adaptive response also includes inhibited glycolysis and extended CCR-mediated carbohydrate-dependent metabolic switches. This makes a great contribution to understanding better the mechanisms of carbon starvation induced stringent response in this important zoonotic pathogen.

## Materials and Methods

### Bacterial strains, plasmids, and growth conditions

All the bacterial strains and plasmids used in this study are listed in Supplementary Table S5. *S. suis* serotype 2 strain SC-19 was isolated from a diseased pig in Sichuan province of China in 2005^4^. *S. suis* was grown in tryptic soy broth (TSB) or on tryptic soy agar (TSA; Difco, France) plates containing 5% newborn bovine serum (Sijiqing, Hangzhou, China). *E. coli* DH5α was cultured in/on Luria-Bertani (LB) broth or plate (Oxoid, Basingstoke, UK). The chemically defined medium (CDM) for *S. suis*^46^ was modified when necessary (see Supplementary Table S6). For ^32^P-labeling *in vivo*, MOPS-CDM (the phosphate compounds-deleted CDM supplemented with 40 mM MOPS, 4 mM Tricine, 0.28 mM K_2_SO_4_ and 50 mM NaCl) was used. When necessary, antibiotics were added to the plate or broth at the following concentrations: 100 μg/ml spectinomycin (Spc), 2.5 μg/ml erythromycin (Erm) for *S. suis*; 50 μg/ml Spc, 180 μg/ml Erm, 50 μg/ml kanamycin (Kan) for *E. coli*.

### Expression and purification of RelA and RelQ

The coding sequences of *relA* and *relQ* were amplified from the genomic DNA of *S. suis* SC-19 using primers relAF/relAR and relQF/relQR (see Supplementary Table S7), respectively. The primers were designed according to the sequences of genes SSU05_2094 and SSU05_1060 of *S. suis* 05ZYH33 (GenBank accession no. CP000407), and cloned into a prokaryotic expression vector pET-28a (Novagen, Shanghai, China), respectively. The resultant plasmids pET28a-*relA* and pET28a-*relQ* were confirmed by DNA sequencing and transformed into *E. coli* BL21(DE3) for expression of His-tagged recombinant proteins, respectively. The bacteria were induced by 1 mM isopropyl-beta-D-thiogalactopyranoside (IPTG) at 37 °C for 3 h. Purification of the recombinant proteins was achieved using Ni-NTA agarose (Bio-Rad, Shanghai, China) under native condition according to the manufacturer’s instructions. Electrophoresis was carried out with 12% SDS-PAGE.

### (p)ppGpp synthetase/hydrolase activity assays

(p)ppGpp synthetase activity of RelA and RelQ assays was performed as described previously^19^. The reaction was carried out at a final volume of 25 μl containing 2 mM ATP, 10 μCi ml^−1^ [γ-P^32^]-ATP, 1.3 mM GTP or GDP and the purified 0.5 μg of recombinant RelA or RelQ in reaction buffer [50 mM Tris-acetate (pH 7.8), 3.3 mM magnesium acetate, 60 mM potassium acetate, 30 mM ammonium acetate, 1 mM dithiothreitol]. The mixture was incubated at 30 °C for 1 h, and the reaction was stopped by adding 1 μl of 88% formic acid. 5 μl of each sample was spotted on polyethyleneimine cellulose plastic-backed Thin-Layer Chromatography (TLC) plate (Merck, Darmstadt Germany) and chromatographed in 1.5 M KH_2_PO_4_ (pH 3.4) for TLC analysis. TLC plates were exposed to a phosphor screen (GE Healthcare, NJ, USA) and radioactivity was scanned by Typhoon FLA 7000 IP (GE Healthcare).

The hydrolase activity assay was performed as described previously with some modification^12^. In brief, hydrolysis reaction mixtures contained 50 mM HEPES (pH 8.0), 150 mM NaCl, 1 mM DTT, 1.4 mM MnCl_2_, 0.7 mM ppGpp (TriLink, San Diego, USA) and 200 nM RelA or RelQ protein. In the negative control, the protein was replaced by *dd*H_2_O. After 5 min reaction at room temperature, the hydrolysis reaction product PPi was detected by measuring the fluorescent product (λ_ex_ = 316/λ_em_ = 456 nm) proportional to the pyrophosphate present using Pyrophosphate Assay Kit according to the product manual (Sigma-Aldrich, St. Louis, USA).

### Construction of mutant strains

To inactivate *relA* gene in *S. suis* strain SC-19, a thermosensitive homologous suicide vector pSET4s::*relA* carrying the left arm (L_arm, 731 bp), right arm (R_arm, 762 bp) and Erm resistance cassette (*erm*^r^) was constructed. The two arms were amplified from the chromosomal DNA of SC-19 using primers relAL01/relAL02 and relAR01/relAR02, respectively. The *erm*^r^ was amplified from the plasmid pAT18 by using primers ermF/ermR (see Supplementary Table S7). The recombinant plasmid pSET4s::*relA* was electro-transformed into SC-19, and the strains were selected on Spc and Erm plates as described previously^47^. The suspected mutant strain Δ*relA* was verified by PCR, RT-PCR and Southern blot analysis using standard protocols. The expression of genes upstream and downstream of *relA* was also tested by RT-PCR. Using similar methods, *relQ* gene was inactivated in strains SC-19 and Δ*relA*, resulting in mutant strains Δ*relQ* and Δ*relA*Δ*relQ*.

### Detection of intracellular (p)ppGpp

*S. suis* strains were grown in TSB with 5% newborn bovine serum as described above. At culture density of OD_600nm_ ≈ 0.2, cells were collected by centrifugation at 3000 *g* for 2 min at room temperature, and the pellets were washed and resuspended in MOPS-CDM to the same density. Then 20 μl of each cell suspension was added into the wells of 96-well microtiter plates and pre-warmed at 37 °C for 10 min. Glucose was the only carbon sources in the CDM, *S. suis* could not grow in the CDM without glucose. According to the previous report with some modification^21^, we used medium with 0.2% glucose to simulate glucose starvation. To simulate glucose starvation, cell suspensions in 0.2% glucose MOPS-CDM were mixed with 130 μl of 0.2% glucose MOPS-CDM containing 150 μCi/ml of H_3_[^32^P]O_4_. Cell suspensions mixed with 1% glucose MOPS-CDM containing 150 μCi/ml of H_3_[^32^P]O_4_ were used as controls. After incubation at 37 °C for 30 min, each 20 μl sample was removed and mixed with an equal volume of 13 M frozen formic acid, then the mixtures were refrozen and thawed twice. 5 μl of each thawed sample was analyzed by TLC as described above. The ppGpp contents of *S. suis* at the time point of sample collection of microarray analysis were further detected by anion exchange HPLC using a Mono Q 5/50 GL column (GE Healthcare) as previously described^16^. ppGpp standard was purchased from TriLink Biosciences (TriLink, San Diego, USA). Standard curves established that the linear range of detection of ppGpp is 50 nM–100 mM.

### Growth in glucose starvation

The growth curves of *S. suis* WT and mutant strains during glucose starvation were tested as described below. Overnight cultured cells, including SC-19, Δ*relA*, Δ*relQ* and Δ*relA*Δ*relQ* strains, were collected by centrifugation at 3000 g for 5 min and washed with CDM for three times. Then the pellets were resuspended in glucose starvation CDM (CDM containing 0.2% glucose) to the density of OD_600_ ≈ 0.1. Cells resuspended in complete CDM (CDM containing 1% glucose) were used as controls. All the samples were cultured at 37 °C, and OD_600nm_ value was measured once each hour.

### Microarray analysis

To identify the genes regulated by RelA/(p)ppGpp during glucose starvation, microarray analysis was performed. Overnight cultured cells were washed with CDM for three times and resuspended in CDM containing 0.2% glucose and CDM containing 1% glucose to the density of OD600 ≈ 0.1 respectively. Cells in early exponential growth period (5 h in Fig. 4) were collected for microarray analysis. The test of each strain included three biological replicates. Briefly, total RNA were isolated and purified using QIAGEN RNeasy Mini Kit (Qiagen, Shanghai, China) according to the manufacturer’s instructions. 2 μg RNA was reverse-transcribed into cDNA, and then the cDNA was transcribed into aaUTP labeled cRNA and purified. 4 μg cRNA was labeled with Cy3 NHS ester (GE healthcare) and purified. After fragmentation of the Cy3-cRNA, hybridization was performed using a Gene Expression Hybridization Kit (Agilent Technologies, Beijing, China). Finally, arrays were scanned by Agilent Microarray Scanner System with resolution of 5 μm. The signal intensities were normalized using Feature Extraction Software (Agilent Technologies) and transformed into log2 values. The microarray data have been submitted to the NCBI Gene Expression Omnibus (GEO) functional genomics data repository under the accession number GSE70092.

### Quantitative RT-PCR (qRT-PCR)

Primers (see Supplementary Table S7) were designed based on the *S. suis* 05ZYH33 genome sequence. RNA extraction was carried out as described in microarray analysis. qRT-PCR was performed on an ABI 7300 HT Sequence Detection System using the ABI Power SYBE Green PCR Master Mix. The expression level of each tested gene was normalized to those of *gapdh*, which did not show any change in expression across all culture condition in both strains SC-19 and Δ*relA*. Data were reported as mean relative expression levels (±standard deviation) under glucose starvation *versus* normal culture condition.

### Statistical analysis

All the experiments were performed at least thrice with triplicate repeats. The means of two groups were compared using Student’s t test (unpaired, 2-tailed), with *p* < 0.05 considered to be statistically significant. Statistical analysis was performed using GraphPad Prism 6 (San Diego, USA). Microarray data were analyzed using GeneSpring Software 5.0 (Silicon Genetics, CA, USA). Those genes with greater than two-fold change ratios were regarded as differentially expressed genes.

## Additional Information

**How to cite this article**: Zhang, T. *et al.* The roles of RelA/(p)ppGpp in glucose-starvation induced adaptive response in the zoonotic *Streptococcus suis*. *Sci. Rep.* **6**, 27169; doi: 10.1038/srep27169 (2016).

## Supplementary Material

Supplementary Information
