# Supplementary material for: The roles of RelA/(p)ppGpp in glucose-starvation induced adaptive response in the zoonotic Streptococcus suis
Source: Sci Rep. 2016 Jun 3;6:27169. doi: 10.1038/srep27169 (PMC4891663; doi:10.1038/srep27169)
Supplement: Supplementary Information [file srep27169-s1.pdf]

Supplementary Information

The roles of RelA/(p)ppGpp in glucose-starvation induced adaptive response in the zoonotic *Streptococcus suis*

Tengfei Zhang,[1, 2, a] Jiawen Zhu,[1, a] Shun Wei,[1] Qingping Luo,[2] Lu Li,[1, 3] Shengqing Li,[4] Alexander Tucker,[5] Huabin Shao, [2] Rui Zhou[1, 3*]

[1] State Key Laboratory of Agricultural Microbiology and Key Laboratory of Veterinary Diagnosis (Ministry of Agriculture), College of Veterinary Medicine, Huazhong Agricultural University, Wuhan 430070, China

[2] Hubei Key Laboratory of Animal Embryo and Molecular Breeding, Institute of Animal and Veterinary Science, Hubei Academy of Agricultural Sciences, Wuhan 430064, China

[3] Cooperative Innovation Center of Sustainable Pig Production, Wuhan 430070, China

[4] Department of Chemistry, College of Science, Huazhong Agricultural University, Wuhan 430070, China

[5] Department of Veterinary Medicine, University of Cambridge, Madingley Road, Cambridge, CB3 0ES, UK

[a] These authors contributed equally to this work

* Corresponding: Prof. Dr. Rui Zhou

Mailing address: State Key Laboratory of Agricultural Microbiology, College of Veterinary Medicine, Huazhong Agricultural University, Shizishan Street 1, Wuhan 430070, China

Phone: +86 27 87281878

Fax: +86 27 87282608

e-mail: rzhou@mail.hzau.edu.cn

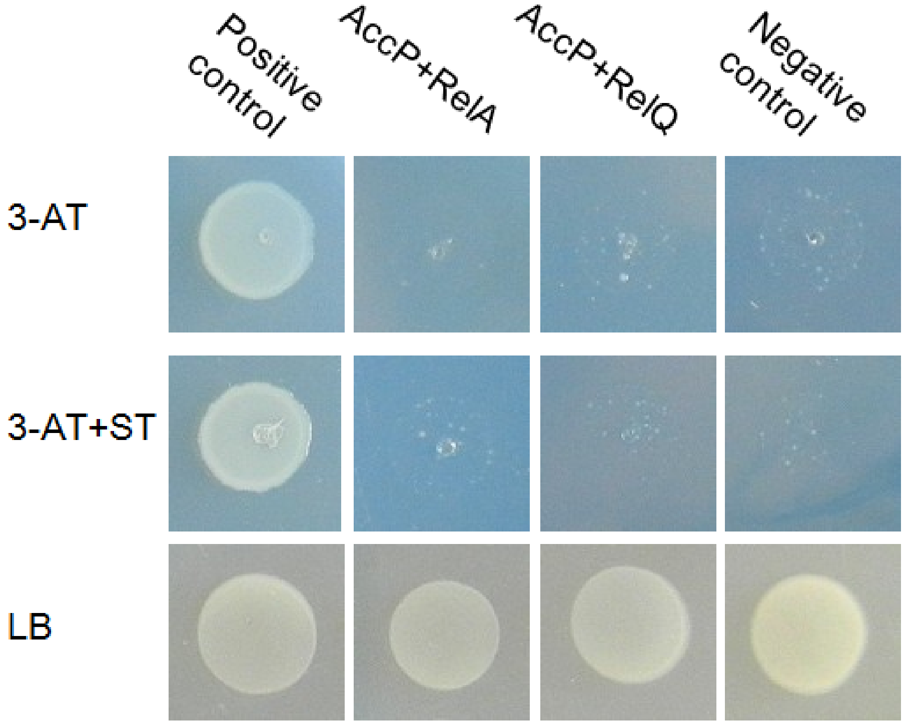

Figure S1. Neither RelA nor RelQ can interact with AccP in *S. suis*. Bacterial Two-Hybrid analysis was carried out using BacterioMatch II Two-Hybrid System Vector Kit (Agilent Technologies, USA). The results showed that strains incorporated neither plasmid with accP+relA nor accP+relQ could grow in screening plates with 3-AT, which suggested that both RelA and RelQ cannot interact with AccP in *S. suis*.

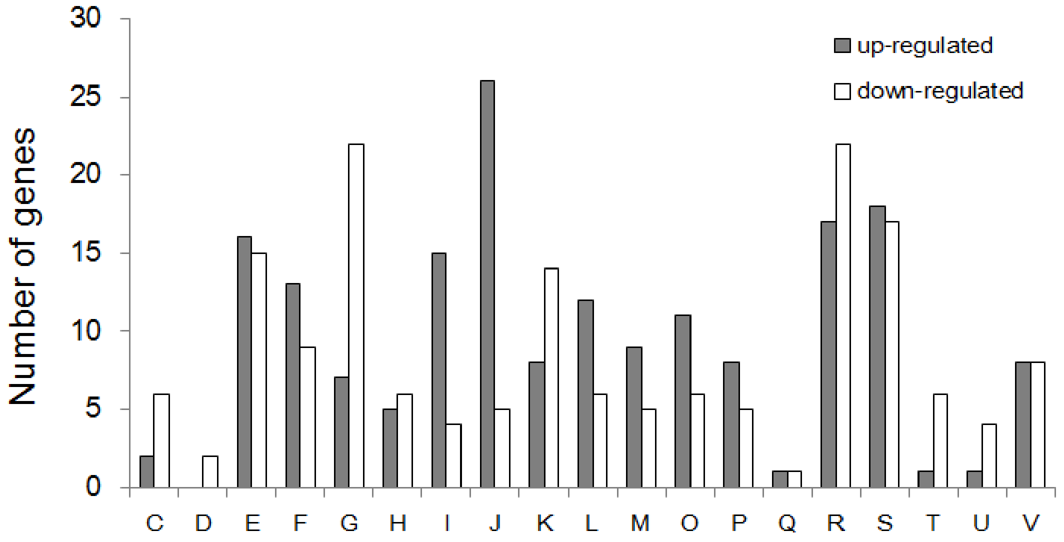

FIG. S2. COG analysis of differential expressed (DE) genes between Δ*relA* and SC-19 in normal condition (CDM containing 1% glucose). C, Energy production and

conversion; D, Cell cycle control, mitosis and meiosis; E, Amino acid transport and metabolism; F, Nucleotide transport and metabolism; G, Carbohydrate transport and metabolism; H, Coenzyme transport and metabolism; I, Lipid transport and metabolism; J, Translation; K, Transcription; L, Replication, recombination and repair; M, Cell wall/membrane biogenesis; O, Posttranslational modification, protein turnover, chaperones; P, Inorganic ion transport and metabolism; Q, Secondary metabolites biosynthesis, transport and catabolism; R, General function prediction only; S, Function unknown; T, Signal transduction mechanisms; U, Intracellular trafficking and secretion; V, Defense mechanisms

Table S1. Comfirmation of microarray results of SC-19 by qRT-PCR

Gene locus	Gene name	Results of microarray	Result of RT-PCR
SSU05_0543	*pfkA*	-3.44	-8.67
SSU05_0336	*fbaA*	-5.36	-56.50
SSU05_0155	*gapA*	-2.74	-5.05
SSU05_1802	*fabF*	+3.07	+1.40
SSU05_2054	*tktA*	-2.96	-4.26
SSU05_1064		-3.07	-40.65
SSU05_0627	*arcC*	+525.14	+50.94

Table S2. Comfirmation of microarray results of Δ*relA* by qRT-PCR

Gene locus	Gene name	Results of microarray	Result of qRT-PCR
SSU05_0543	*pfkA*	-1.74	-1.37
SSU05_0157	*pgk*	-1.05	-1.70
SSU05_0155	*gapA*	-1.39	-1.30
SSU05_1807	*fabH*	-9.06	-26.52
SSU05_2054	*tktA*	-2.96	-4.26
SSU05_1372	*ccpA*	-4.02	-2.34
SSU05_0627	*arcC*	+305.11	+17.38

Table S3. The regulated genes in SC-19 during glucose starvation

Amino acid transport and metabolism

Upregulated

ID	FC	Description
SSU05_0349	3.8977	aminotransferase AlaT
SSU05_0557	5.43	gamma-glutamyl kinase
SSU05_0558	3.3174	gamma-glutamyl phosphate reductase
SSU05_0613	4.7332	aspartate aminotransferase

SSU05_0624	64.3563	arginine deiminase
SSU05_0626	59.0989	ornithine carbamoyltransferase
SSU05_0627	525.1393	carbamate kinase
SSU05_0629	47.4398	hypothetical protein
SSU05_0852	2.5282	serine hydroxymethyltransferase
SSU05_1029	4.8779	ABC-type polar amino acid transport system, ATPase component
SSU05_1033	2.5488	O-acetylhomoserine sulfhydrylase
SSU05_1363	4.8234	amino acid ABC transporter, permease protein
SSU05_1365	6.1128	ABC-type amino acid transport system, permease component
SSU05_1387	27.7874	amylase-binding protein B
SSU05_1561	2.3521	bifunctional beta-cystathionase/maltose regulon repressor
SSU05_1562	2.1188	cystathionine gamma-synthase
SSU05_1774	2.65	5-methyltetrahydropteroyltriglutamate--homocysteine S-methyltransferase
SSU05_1775	3.1209	5,10-methylenetetrahydrofolate reductase
SSU05_1882	2.5584	amino acid ABC transporter ATP-binding protein
SSU05_1885	2.1451	threonine dehydratase
SSU05_1946	3.4081	isopropylmalate isomerase small subunit
SSU05_1947	5.5759	3-isopropylmalate dehydratase large subunit
SSU05_2017	3.4762	argininosuccinate synthase
SSU05_2067	4.1056	putative amino acid ABC transporter, ATP-binding protein
SSU05_2068	4.7643	ABC-type amino acid transport system, permease component
SSU05_1388	3.1224	threonine dehydrogenase and related Zn-dependent dehydrogenase
SSU05_0667	2.4415	phosphoglycerate dehydrogenase and related dehydrogenase
SSU05_1886	2.763	ketol-acid reductoisomerase
SSU05_0791	3.6922	carbamoyl phosphate synthase small subunit
SSU05_0719	2.1974	dihydrodipicolinate synthase
SSU05_1948	4.5206	3-isopropylmalate dehydrogenase
SSU05_1362	2.9685	glutamine ABC transporter substrate-binding protein
SSU05_0562	3.1206	hypothetical protein
**downregulated**		
ID	FC	Description
SSU05_0022	2.8497	aromatic amino acid aminotransferase
SSU05_0174	4.6694	putative integral membrane protein; branched-chain amino acid permease
SSU05_0494	6.5298	ABC-type amino acid transport system, permease component
SSU05_0495	4.0599	ABC-type amino acid transport system, permease component
SSU05_0496	2.0535	putative amino acid ABC transporter, ATP-binding protein
SSU05_0547	4.2505	lysophospholipase L1 and related esterase
SSU05_0550	10.1231	amino acid (glutamine) ABC transporter, permease protein
SSU05_0551	5.1558	ABC-type polar amino acid transport system, ATPase component

SSU05_0675	10.5094	amino acid transporter
SSU05_0774	8.507	threonine aldolase
SSU05_0776	9.2989	cysteine sulfinate desulfinase/cysteine desulfurase
SSU05_0778	40.6478	branched-chain amino acid permease
SSU05_0780	2.3457	branched-chain amino acid permease
SSU05_0803	2.373	homoserine kinase
SSU05_0805	2.9467	ABC-type spermidine/putrescine transport system, ATPase component
SSU05_0806	2.3246	ABC-type spermidine/putrescine transport system, permease component I
SSU05_0808	2.4035	spermidine/putrescine-binding periplasmic protein
SSU05_0828	4.4426	dihydrodipicolinate reductase
SSU05_1030	2.8625	histone acetyltransferase HPA2-like acetyltransferase
SSU05_1066	19.2999	cysteine sulfinate desulfinase/cysteine desulfurase
SSU05_1243	2.1706	oligoendopeptidase F
SSU05_1306	7.1362	homoserine O-succinyltransferase
SSU05_1343	6.191	lactoylglutathione lyase and related lyases
SSU05_1546	2.7343	ABC-type branched-chain amino acid transport system, permease component
SSU05_1547	4.7141	branched chain amino acid ABC transporter permease
SSU05_1548	3.1748	hypothetical protein
SSU05_1649	6.4381	lysophospholipase L1 and related esterases
SSU05_1770	3.4872	acetylornithine deacetylase/succinyl-diaminopimelate desuccinylase-like protein
SSU05_1811	5.3538	aspartate kinase
SSU05_1825	2.4176	aminopeptidase P; XAA-pro aminopeptidase
SSU05_1881	6.0584	amino acid ABC transporter periplasmic protein
SSU05_2047	4.9956	2,3,4,5-tetrahydropyridine-2-carboxylate N-succinyltransferase, putative
SSU05_0665	2.6448	phosphoserine aminotransferase
SSU05_1889	2.9146	thiamine pyrophosphate-requiring enzyme
SSU05_1867	4.0653	ABC-type dipeptide/oligopeptide/nickel transport system, ATPase component
SSU05_1065	13.4083	ribose-phosphate pyrophosphokinase
SSU05_0552	2.9937	amino acid ABC transporter, amino acid-binding protein
SSU05_1017	13.5542	amino acid ABC transporter periplasmic protein
SSU05_1018	3.3965	putative amino acid transporter, amino acid-binding protein
SSU05_1026	3.0263	amino acid ABC transporter periplasmic protein
SSU05_1880	5.2855	hypothetical protein

Carbohydrate transport and metabolism

upregulated

ID	FC	Description
SSU05_0168	65.0235	ABC-type sugar transport system, periplasmic component
SSU05_0169	101.5793	ABC-type sugar transport system, periplasmic component

SSU05_0170	60.7185	ABC-type sugar transport system, permease component
SSU05_0171	66.5909	ABC-type sugar transport system, permease component
SSU05_0172	33.0629	Alpha-galactosidase
SSU05_0184	32.2177	Beta-glucosidase/6-phospho-beta-glucosidase/beta-galactosidase
SSU05_0185	5.0177	Beta-glucosidase/6-phospho-beta-glucosidase/beta-galactosidase
SSU05_0188	46.3954	phosphotransferase system, galactitol-specific IIB component
SSU05_0205	3.4428	fructose-1-phosphate kinase-like protein
SSU05_0206	3.1298	fructose-1-phosphate kinase-like protein
SSU05_0230	88.5056	glycosidase
SSU05_0231	57.1092	phosphotransferase system IIC component, glucose/maltose/N-acetylglucosamine-specific
SSU05_0360	50.1601	galactokinase
SSU05_0361	38.1407	galactose-1-phosphate uridylyltransferase
SSU05_0398	4.5719	phosphotransferase system IIC component, glucose/maltose/N-acetylglucosamine-specific
SSU05_0449	104.7061	Beta-galactosidase
SSU05_0450	110.127	phosphotransferase system, mannose/fructose/N-acetylgalactosamine-specific component IIB
SSU05_0451	708.8139	phosphotransferase system, mannose/fructose/N-acetylgalactosamine-specific component IIC
SSU05_0452	448.2165	phosphotransferase system, mannose/fructose/N-acetylgalactosamine-specific component IID
SSU05_0453	28.6443	phosphotransferase system, mannose/fructose-specific component IIA
SSU05_0454	16.909	galactose mutarotase-like protein
SSU05_0630	2.5216	N-acetyl-beta-hexosaminidase
SSU05_0686	5.1604	phosphomannomutase
SSU05_0823	9.9511	fructose-1-phosphate kinase-like protein
SSU05_0824	18.6936	phosphotransferase system, fructose-specific IIC component
SSU05_0825	18.2157	phosphotransferase system, fructose-specific IIC component
SSU05_0882	3.5484	phosphomannomutase
SSU05_1013	25.0685	ADP-glucose pyrophosphorylase
SSU05_1014	34.0745	ADP-glucose pyrophosphorylase
SSU05_1015	26.6331	glycogen synthase
SSU05_1016	18.8722	1,4-alpha-glucan branching enzyme
SSU05_1035	4.0719	galactose mutarotase-like protein
SSU05_1153	22.4207	Beta-glucosidase-related glycosidase
SSU05_1154	71.5797	hypothetical protein

SSU05_1157	112.4177	mannonate dehydratase
SSU05_1158	99.9815	glucuronate isomerase
SSU05_1159	49.2005	glucuronate isomerase
SSU05_1160	26.9941	2-keto-3-deoxy-6-phosphogluconate aldolase
SSU05_1217	109.1796	phosphotransferase system, mannose/fructose/N-acetylgalactosamine-specific component IID
SSU05_1218	67.2253	phosphotransferase system, mannose/fructose/N-acetylgalactosamine-specific component IIC
SSU05_1219	102.0855	phosphotransferase system, mannose/fructose/N-acetylgalactosamine-specific component IIB
SSU05_1221	5.4676	phosphotransferase system, mannose/fructose-specific component IIA
SSU05_1222	5.7705	keto-hydroxyglutarate-aldolase/keto-deoxy-phosphogluconate aldolase
SSU05_1223	3.9748	ribokinase family sugar kinase
SSU05_1224	6.7385	hypothetical protein
SSU05_1258	4.7573	tagatose 1,6-diphosphate aldolase
SSU05_1259	12.8525	N-acetylglucosamine-6-phosphate deacetylase
SSU05_1314	2.2785	HrpA-like helicase
SSU05_1336	21.7917	Beta-fructosidases (levanase/invertase)
SSU05_1337	22.2765	Beta-fructosidases (levanase/invertase)
SSU05_1338	48.0138	ABC-type sugar transport system, periplasmic component
SSU05_1339	60.0061	ABC-type sugar transport system, permease component
SSU05_1340	34.2725	ABC-type polysaccharide transport system, permease component
SSU05_1401	12.0144	phosphotransferase system IIC component, glucose/maltose/N-acetylglucosamine-specific
SSU05_1444	2.1308	maltodextrin phosphorylase
SSU05_1448	2.3995	phosphopentomutase
SSU05_1449	2.6458	ribose-5-phosphate isomerase A
SSU05_1554	4.2946	glycosidase
SSU05_1555	4.5092	glycosidase
SSU05_1560	6.8984	Alpha-galactosidase
SSU05_1635	2.0666	xylanase/chitin deacetylase
SSU05_1779	4.3848	mannose-specific PTS IIC
SSU05_1780	8.9392	mannose-specific PTS IID
SSU05_1817	14.1481	phosphotransferase system IIC component, glucose/maltose/N-acetylglucosamine-specific
SSU05_1818	6.7551	Beta-fructosidases (levanase/invertase)
SSU05_1907	13.5036	ABC-type sugar transport system, ATPase component
SSU05_1914	73.2162	ABC-type sugar transport system, periplasmic component

SSU05_1915	28.952	ABC transporter membrane spanning permease - sugar transport
SSU05_1916	23.0019	ABC transporter, permease protein
SSU05_1919	2.8895	hypothetical protein
SSU05_1921	25.9117	putative alpha-1,2-mannosidase
SSU05_1922	2.1119	endo-beta-N-acetylglucosaminidase, putative
SSU05_1931	6.3294	pts system, sucrose-specific IIBC component
SSU05_1957	81.1603	dihydroxyacetone kinase
SSU05_1958	92.9864	dihydroxyacetone kinase
SSU05_1960	41.8946	glycerol uptake facilitator and related permease (major Intrinsic protein family)
SSU05_2062	3.001	phosphotransferase system, galactitol-specific IIB component
SSU05_2073	27.8847	phosphotransferase system cellobiose-specific component IIA
SSU05_2074	24.9612	phosphotransferase system cellobiose-specific component IIA
SSU05_2075	23.0873	phosphotransferase system cellobiose-specific component IIB
SSU05_2078	7.2257	Beta-glucosidase/6-phospho-beta-glucosidase/beta-galactosidase
SSU05_2079	5.4473	alpha-galactosidase/6-phospho-beta-glucosidase
SSU05_2080	5.5356	alpha-galactosidase/6-phospho-beta-glucosidase
SSU05_2131	8.248	4-alpha-glucanotransferase
SSU05_2132	9.7267	4-alpha-glucanotransferase
SSU05_2133	5.3656	ABC transporter substrate-binding protein - maltose/maltodextrin
SSU05_2134	5.4325	ABC-type sugar transport system, permease component
SSU05_2135	5.9093	ABC-type maltose transport system, permease component
SSU05_2138	4.7646	Type II secretory pathway, pullulanase PulA and related glycosidases
SSU05_2147	2.9344	Butyrivibrio fibrisolvens sp|P16084|BGLS_BUTFI Beta-glucosidase A (Gentiobiase) (Cellobiase) (Beta-D
SSU05_2071	38.9867	phosphotransferase system cellobiose-specific component IIC
SSU05_0822	3.949	sugar metabolism transcriptional regulator
SSU05_0832	2.8729	transcriptional regulator/sugar kinase
**downregulated**		
ID	FC	Description
SSU05_0155	2.7363	glyceraldehyde-3-phosphate dehydrogenase
SSU05_0268	33.4359	hypothetical protein
SSU05_0336	5.36	fructose/tagatose bisphosphate aldolase
SSU05_0337	6.2107	fructose-bisphosphate aldolase class-II
SSU05_0338	4.4062	fructose-bisphosphate aldolase

SSU05_0339	3.8812	fructose/tagatose bisphosphate aldolase
SSU05_0520	2.6916	fructose-2,6-bisphosphatase
SSU05_0531	14.2723	triosephosphate isomerase
SSU05_0543	3.4415	6-phosphofructokinase
SSU05_0544	2.4957	pyruvate kinase
SSU05_0634	7.3426	6-phosphogluconolactonase/glucosamine-6-phosphate isomerase/deaminase
SSU05_0802	3.2567	xylanase/chitin deacetylase
SSU05_1059	2.2547	inorganic polyphosphate/ATP-NAD kinase
SSU05_1109	3.6862	hypothetical protein
SSU05_1436	2.8141	HAD family sugar phosphatase
SSU05_1497	5.4336	3-carboxymuconate cyclase
SSU05_1503	6.4696	putative enolase
SSU05_1638	5.0227	phosphoglycerate mutase 1
SSU05_2054	2.9607	transketolase
SSU05_2064	2.2695	Type II secretory pathway, pullulanase PulA and related glycosidases
SSU05_0567	2.6548	Cps2D
SSU05_1286	2.5786	ABC-type polysaccharide/polyol phosphate transport system, ATPase component
SSU05_1287	2.0889	ABC-type polysaccharide/polyol phosphate export system, permease component
SSU05_0706	4.8847	sugar metabolism transcriptional regulator
SSU05_1045	11.349	sugar metabolism transcriptional regulator

Cell cycle control, mitosis and meiosis

upregulated

ID	FC	Description
SSU05_0104	3.4168	hypothetical protein

downregulated

ID	FC	Description
SSU05_0010	2.268	septum formation initiator
SSU05_0013	4.3604	tRNA(Ile)-lysidine synthase
SSU05_0481	2.7905	cell division protein FtsZ
SSU05_0487	3.4936	cell division initiation protein
SSU05_0526	2.3781	cell division membrane protein
SSU05_0566	2.2193	Cps2C
SSU05_1410	3.3872	cell division protein
SSU05_1411	3.3346	ATPase involved in cell division
SSU05_1512	6.6799	rod shape determining protein
SSU05_1743	21.0049	protein required for the initiation of cell division
SSU05_2004	5.4527	antitoxin of toxin-antitoxin stability system
SSU05_0344	4.0129	L-asparaginase/ Glu-tRNAGln amidotransferase subunit D
SSU05_0345	2.7549	L-asparaginase/ Glu-tRNAGln amidotransferase subunit D
SSU05_1500	5.1633	cytotoxic translational repressor of toxin-antitoxin stability

		system
SSU05_0886	4.2275	hypothetical protein

Cell wall/membrane biogenesis

upregulated

ID	FC	Description
SSU05_0195	8.8782	phosphosugar isomerase
SSU05_0254	18.5984	hypothetical protein
SSU05_1292	2.3452	phosphoglycerol transferase/alkaline phosphatase superfamily protein
SSU05_1998	2.1797	phosphoglycerol transferase/alkaline phosphatase superfamily protein
SSU05_2103	2.7019	cell wall anchor domain-containing protein
SSU05_0719	2.1974	dihydrodipicolinate synthase

downregulated

ID	FC	Description
SSU05_0058	6.4876	Heme/copper-type cytochrome/quinol oxidase, subunit 1
SSU05_0266	8.1227	putative effector of murein hydrolase
SSU05_0267	9.2232	putative effector of murein hydrolase
SSU05_0329	9.9763	hypothetical protein
SSU05_0414	3.1487	membrane carboxypeptidase (penicillin-binding protein)
SSU05_0565	2.2649	Cps2B
SSU05_0568	2.745	Cps2E
SSU05_0569	2.4061	Cps2F
SSU05_0570	2.7809	glycosyltransferase
SSU05_0573	2.1604	Cps2J
SSU05_0641	2.9604	hypothetical protein
SSU05_0804	42.1381	UDP-N-acetylenolpyruvoylglucosamine reductase
SSU05_1074	4.8653	sortase
SSU05_1275	3.6994	cell wall biosynthesis glycosyltransferase
SSU05_1277	4.7277	cell wall biosynthesis glycosyltransferase
SSU05_1278	4.2106	glycosyltransferase
SSU05_1279	3.246	lipopolysaccharide biosynthesis protein
SSU05_1285	2.6218	polysaccharide biosynthesis protein
SSU05_1288	5.2912	polysaccharide biosynthesis protein/putative rhamnosyl transferase
SSU05_1290	7.611	glycosyltransferase
SSU05_1350	2.9807	UDP-N-acetylmuramyl pentapeptide synthase
SSU05_1354	2.5206	cell division protein FtsI/penicillin-binding protein 2
SSU05_1430	9.3084	large-conductance mechanosensitive channel
SSU05_1431	2.753	UDP-glucose 4-epimerase
SSU05_1432	4.0044	UDP-glucose 4-epimerase
SSU05_1619	2.3842	UDP-N-acetylglucosamine 1-carboxyvinyltransferase
SSU05_1720	3.5665	UDP-N-acetylmuramate--L-alanine ligase
SSU05_1741	4.6428	phospho-N-acetylmuramoyl-pentapeptide-transferase

SSU05_1742	3.1483	cell division protein FtsI/penicillin-binding protein 2
SSU05_1744	31.7966	S-adenosyl-methyltransferase MraW
SSU05_1852	3.0449	hypothetical protein
SSU05_1870	2.3888	D-alanyl-D-alanine carboxypeptidase
SSU05_1877	4.6936	UDP-N-acetylmuramyl pentapeptide phosphotransferase/UDP-N-acetylglucosamine-1-phosphate transferase
SSU05_1985	2.433	penicillin-binding protein 2A
SSU05_2041	2.2327	glucose-1-phosphate-uridylyltransferase
SSU05_2144	2.1729	cell wall biosynthesis glycosyltransferase
SSU05_2173	16.491	FOG: LysM repeat
SSU05_0567	2.6548	Cps2D
SSU05_1286	2.5786	ABC-type polysaccharide/polyol phosphate transport system, ATPase component
SSU05_1287	2.0889	ABC-type polysaccharide/polyol phosphate export system, permease component
SSU05_0785	7.0739	prolipoprotein signal peptidase; Lsp

Coenzyme transport and metabolism

upregulated

ID	FC	Description
SSU05_0312	2.1462	phosphomethylpyrimidine kinase
SSU05_0369	3.0174	nicotinic acid mononucleotide adenylyltransferase
SSU05_0370	3.3911	HD superfamily hydrolase
SSU05_0733	2.6626	phosphomethylpyrimidine kinase
SSU05_0734	2.4389	hydroxyethylthiazole kinase
SSU05_0956	2.6721	hypothetical protein
SSU05_1088	2.1755	pantothenate kinase
SSU05_1755	2.2784	geranylgeranyl pyrophosphate synthase
SSU05_1756	2.2019	geranylgeranyl pyrophosphate synthase
SSU05_1967	2.1763	bifunctional glutamate--cysteine ligase/glutathione synthetase
SSU05_0667	2.4415	phosphoglycerate dehydrogenase and related dehydrogenase
SSU05_1886	2.763	ketol-acid reductoisomerase

downregulated

ID	FC	Description
SSU05_0056	2.7979	folylpolyglutamate synthase
SSU05_0497	3.2283	5,10-methylene-tetrahydrofolate dehydrogenase/Methenyl tetrahydrofolate cyclohydrolase
SSU05_0689	9.9658	phosphopantothenate--cysteine ligase
SSU05_0777	3.0646	thiamine biosynthesis protein ThiI
SSU05_0835	25.7437	dihydrofolate reductase
SSU05_1113	6.0321	bifunctional riboflavin kinase/FMN adenylyltransferase
SSU05_1141	2.5795	putative 2-amino-4-hydroxy-6-hydroxymethylpteridine pyrophosphokinase

SSU05_1144	28.242	GTP cyclohydrolase I
SSU05_1441	2.4352	coproporphyrinogen III oxidase
SSU05_1466	6.233	SAM-dependent methyltransferase
SSU05_1673	6.7752	NAD synthetase
SSU05_1680	10.0079	phosphopantetheine adenylyltransferase
SSU05_1973	3.0208	hypothetical protein
SSU05_1974	3.1556	transcriptional regulator
SSU05_1992	2.043	thiamine pyrophosphokinase
SSU05_0650	4.7683	ABC-type cobalamin/Fe3+-siderophores transport system, ATPase component
SSU05_0665	2.6448	phosphoserine aminotransferase
SSU05_1889	2.9146	thiamine pyrophosphate-requiring enzyme

Defense mechanisms

up

ID	FC	Description
SSU05_0288	2.3292	Na+-driven multidrug efflux pump
SSU05_0293	2.4612	ABC-type multidrug transport system, ATPase and permease component
SSU05_0294	2.1229	hypothetical protein
SSU05_0540	3.25	ABC-type multidrug transport system, ATPase component
SSU05_0618	2.8247	Na+-driven multidrug efflux pump
SSU05_0748	3.9212	ABC-type multidrug transport system, ATPase component
SSU05_0799	4.9509	ABC-type multidrug transport system, ATPase and permease component
SSU05_0800	3.1495	ABC-type multidrug transport system, ATPase and permease component
SSU05_0891	2.726	peptide ABC transporter ATPase
SSU05_0946	4.1913	hypothetical protein
SSU05_0947	3.7179	ABC-type multidrug transport system, ATPase and permease component
SSU05_0948	4.7793	cytolysin B transport protein
SSU05_1054	2.7096	ABC-type multidrug transport system, ATPase and permease component
SSU05_1381	6.0328	peptide ABC transporter ATPase
SSU05_1406	3.0792	ABC-type multidrug transport system, ATPase and permease component
SSU05_1594	2.2924	peptide ABC transporter ATPase
SSU05_1688	3.6424	putative ATPase
SSU05_1784	2.9689	Type I restriction-modification system methyltransferase subunit
SSU05_1785	3.7229	Type I restriction-modification system methyltransferase subunit
SSU05_1786	3.5587	restriction endonuclease S subunit
SSU05_1787	9.3323	Type I site-specific restriction-modification system, R

		(restriction) subunit and related helicase
SSU05_1056	2.0661	ABC-type multidrug transport system, ATPase and permease component

downregulated

ID	FC	Description
SSU05_0012	4.9799	Beta-lactamase class A
SSU05_0695	2.1905	putative HsdM
SSU05_0696	2.2313	putative HsdS
SSU05_0880	3.5688	hypothetical protein
SSU05_1450	7.7169	Type I restriction enzyme EcoKI specificity protein (S protein)
SSU05_1451	7.1502	type I restriction-modification system, S subunit
SSU05_1674	2.3189	glycopeptide antibiotics resistance protein
SSU05_1855	4.7354	ABC-type multidrug transport system, ATPase component
SSU05_1879	6.3619	undecaprenyl pyrophosphate phosphatase
SSU05_2037	2.5674	ABC-type multidrug transport system, ATPase and permease component

Energy production and conversion

upregulated

ID	FC	Description
SSU05_0200	6.3983	pyruvate-formate lyase
SSU05_0280	18.1492	bifunctional acetaldehyde-CoA/alcohol dehydrogenase
SSU05_0292	2.1041	isopentenyl pyrophosphate isomerase
SSU05_0322	4.1872	NADH:flavin oxidoreductase
SSU05_0518	4.1026	coenzyme F420-dependent N5,N10-methylene tetrahydromethanopterin reductase-like protein
SSU05_0717	8.4541	glycerol dehydrogenase
SSU05_1008	2.7734	hypothetical protein
SSU05_1202	3.6874	isocitrate dehydrogenase
SSU05_1839	2.592	branched-chain alpha-keto acid dehydrogenase subunit E2
SSU05_1948	4.5206	3-isopropylmalate dehydrogenase
SSU05_0319	2.5903	NADPH:quinone reductase and related Zn-dependent oxidoreductase
SSU05_0283	2.5742	ABC-type transport system involved in cytochrome bd biosynthesis, ATPase and permease component

downregulated

ID	FC	Description
SSU05_0135	5.9798	acetate kinase
SSU05_0511	2.0057	hypothetical protein
SSU05_0716	3.3974	glycerol dehydrogenase and related enzyme
SSU05_1076	6.2445	L-lactate dehydrogenase
SSU05_1175	2.8954	mitochondrial oligomycin sensitivity protein
SSU05_1176	4.1735	F0F1-type ATP synthase, subunit b
SSU05_1177	9.2485	F0F1-type ATP synthase, subunit a

SSU05_1178	16.3753	F0F1 ATP synthase subunit C
SSU05_1534	9.9027	putative flavodoxin
SSU05_2040	2.4955	NAD(P)H-dependent glycerol-3-phosphate dehydrogenase
SSU05_2153	2.7968	succinate dehydrogenase/fumarate reductase, flavoprotein subunit
SSU05_2154	5.776	succinate dehydrogenase/fumarate reductase, flavoprotein subunit
SSU05_0140	15.0513	Thiol-disulfide isomerase and thioredoxin

Inorganic ion transport and metabolism

upregulated

ID	FC	Description
SSU05_0115	16.296	copper chaperone
SSU05_0217	3.8195	ABC-type nitrate/sulfonate/bicarbonate transport system, ATPase component
SSU05_0221	2.4964	cation transport ATPase
SSU05_0222	54.467	cation transport ATPase
SSU05_0309	7.828	cation transport ATPase
SSU05_0646	4.3043	ABC-type Fe3+-siderophore transport system, permease component
SSU05_0670	21.3495	Co/Zn/Cd cation transporter
SSU05_0740	6.3468	cobalt ABC transporter permease protein
SSU05_0741	3.9001	cobalt ABC transporter ATP-binding protein
SSU05_0809	3.7834	chloride channel protein EriC
SSU05_0977	2.5908	arsenate reductase
SSU05_1318	2.86	cyanate permease
SSU05_1384	72.9899	cation transport ATPase
SSU05_1385	56.4708	cation transport ATPase
SSU05_1386	65.268	cation transport ATPase
SSU05_1409	2.4915	Fe2+ transport system protein B
SSU05_1768	2.48	ABC-type metal ion transport system, permease component
SSU05_2083	242.2664	zinc ABC transporter, permease protein
SSU05_2084	105.241	Mn2+/Zn2+ ABC transporter permease
SSU05_2085	71.491	unknown pir||T45470
SSU05_2086	35.9013	high-affinity zinc uptake system protein znuA precursor

downregulated

ID	FC	Description
SSU05_0269	3.8069	formate/nitrate transporter
SSU05_0410	2.0077	ABC-type cobalt transport system, permease component CbiQ and related transporters
SSU05_0879	5.5875	adenylylsulfate kinase-like kinase
SSU05_1105	5.5673	ABC-type phosphate transport system, periplasmic component
SSU05_1106	11.532	ABC-type phosphate transport system, periplasmic component

SSU05_1111	3.2987	transcriptional regulator Spx
SSU05_1302	4.6909	divalent heavy-metal cations transporter
SSU05_1537	3.3204	hypothetical protein
SSU05_1539	10.7366	manganese-dependent superoxide dismutase
SSU05_1683	3.0212	rhodanese-related sulfurtransferase
SSU05_1759	2.3722	Trk family potassium uptake protein
SSU05_2032	4.1079	hypothetical protein
SSU05_2174	7.2383	ABC-type cobalt transport system, permease component CbiQ and related transporters
SSU05_2175	5.3717	cobalt transporter ATP-binding subunit
SSU05_2176	7.4004	cobalt transporter ATP-binding subunit
SSU05_1867	4.0653	ABC-type dipeptide/oligopeptide/nickel transport system, ATPase component
SSU05_0650	4.7683	ABC-type cobalamin/Fe3+-siderophores transport system, ATPase component

Intracellular trafficking and secretion

upregulated

ID	FC	Description
SSU05_0969	2.0557	Type IV secretory pathway, VirB4 component
SSU05_1216	154.9073	preprotein translocase subunit YajC

downregulated

ID	FC	Description
SSU05_0913	5.1044	chromosome segregation ATPase
SSU05_1392	3.0337	preprotein translocase subunit SecG
SSU05_1854	3.3591	ABC transporter permease
SSU05_1965	4.9625	hypothetical protein
SSU05_2014	14.0042	hypothetical protein
SSU05_1550	3.8232	ATP-dependent Clp protease proteolytic subunit
SSU05_1551	19.8806	putative ATP-dependent Clp protease, proteolytic subunit
SSU05_0785	7.0739	prolipoprotein signal peptidase; Lsp

Lipid transport and metabolism

upregulated

ID	FC	Description
SSU05_0289	5.7064	mevalonate kinase
SSU05_0290	4.684	mevalonate pyrophosphate decarboxylase
SSU05_0291	2.466	mevalonate kinase
SSU05_1440	2.2456	Acyl-ACP thioesterase
SSU05_1796	2.4566	acetyl-CoA carboxylase subunit alpha
SSU05_1797	2.2439	Acetyl-CoA carboxylase beta subunit
SSU05_1798	3.3895	Acetyl-CoA carboxylase beta subunit
SSU05_1799	2.5806	acetyl-CoA carboxylase biotin carboxylase subunit
SSU05_1800	4.1995	3-hydroxymyristoyl/3-hydroxydecanoyl-(acyl carrier protein) dehydratase
SSU05_1801	3.2543	acetyl-CoA carboxylase biotin carboxyl carrier protein subunit

SSU05_1804	2.0983	(acyl-carrier-protein) S-malonyltransferase
SSU05_1802	3.0731	3-oxoacyl-(acyl carrier protein) synthase II
SSU05_1156	67.2471	D-mannonate oxidoreductase
SSU05_1225	6.3536	gluconate 5-dehydrogenase
SSU05_1587	6.6311	3-ketoacyl-(acyl-carrier-protein) reductase
SSU05_1803	2.6812	3-ketoacyl-(acyl-carrier-protein) reductase
**downregulated**		
ID	FC	Description
SSU05_0652	23.7292	1-acyl-sn-glycerol-3-phosphate acyltransferase
SSU05_1640	2.3319	Acetyl-CoA acetyltransferase
SSU05_1807	2.7159	3-oxoacyl-(acyl carrier protein) synthase III
SSU05_1963	4.3914	CDP-diglyceride synthetase
SSU05_1964	10.1694	undecaprenyl pyrophosphate synthase
SSU05_2177	5.7301	CDP-diacylglycerol--glycerol-3-phosphate 3-phosphatidyltransferase
SSU05_0003	27.9978	sphingosine kinase and enzymes related to diacylglycerol kinase
SSU05_1806	2.1844	acyl carrier protein
SSU05_0886	4.2275	cytotoxic translational repressor of toxin-antitoxin stability system
**Nucleotide transport and metabolism**		
**upregulated**		
ID	FC	Description
SSU05_0033	20.6816	phosphoribosylamine--glycine ligase
SSU05_0034	21.3194	phosphoribosylcarboxyaminoimidazole (NCAIR) mutase
SSU05_0035	12.0297	phosphoribosylaminoimidazole carboxylase ATPase subunit
SSU05_0737	4.2679	uridine phosphorylase
SSU05_0738	5.7391	uridine phosphorylase
SSU05_1000	11.1969	putative 5'-nucleotidase
SSU05_1007	3.3819	orotate phosphoribosyltransferase
SSU05_1009	3.0698	orotidine 5'-phosphate decarboxylase
SSU05_1538	3.6363	putative 5'-nucleotidase
SSU05_1622	2.8593	deoxycytidylate deaminase
SSU05_2095	41.5461	bifunctional 2',3'-cyclic nucleotide 2'-phosphodiesterase/3'-nucleotidase precursor protein
SSU05_2183	6.0925	inosine 5'-monophosphate dehydrogenase
SSU05_0791	3.6922	carbamoyl phosphate synthase small subunit
**downregulated**		
ID	FC	Description
SSU05_0014	6.0287	hypoxanthine-guanine phosphoribosyltransferase
SSU05_0091	8.4457	adenylate kinase
SSU05_0491	2.1547	MutT family hydrolase
SSU05_0661	27.129	thymidylate kinase
SSU05_0690	2.6696	formate--tetrahydrofolate ligase

SSU05_0789	5.4744	bifunctional pyrimidine regulatory protein PyrR uracil phosphoribosyltransferase
SSU05_0815	63.5618	guanosine 5'-monophosphate oxidoreductase
SSU05_0834	16.5463	thymidylate synthase
SSU05_0846	5.253	thymidine kinase
SSU05_0847	10.4746	thymidine kinase
SSU05_1020	2.7189	NTP pyrophosphohydrolase including oxidative damage repair enzymes
SSU05_1145	15.8209	NTP pyrophosphohydrolase including oxidative damage repair enzymes
SSU05_1307	8.6392	adenine phosphoribosyltransferase
SSU05_1327	5.0228	uridylate kinase
SSU05_1553	3.5637	uracil phosphoribosyltransferase
SSU05_1966	2.4136	adenylosuccinate synthase
SSU05_2139	2.2923	ribonucleotide reduction protein
SSU05_2166	2.38	MutT/NudX family protein (putative)
SSU05_1065	13.4083	ribose-phosphate pyrophosphokinase

Posttranslational modification, protein turnover, chaperones

upregulated

ID	FC	Description
SSU05_0149	3.42	GroEL
SSU05_0153	3.7426	metalloendopeptidase
SSU05_0299	39.8262	molecular chaperone GrpE (heat shock protein)
SSU05_0300	26.8145	molecular chaperone DnaK
SSU05_0302	7.4681	DnaJ-like molecular chaperone
SSU05_0389	167.3344	ATPases with chaperone activity, ATP-binding subunit
SSU05_0390	119.6911	ATPases with chaperone activity, ATP-binding subunit
SSU05_0391	113.6877	ATPases with chaperone activity, ATP-binding subunit
SSU05_0492	2.1405	ATPases with chaperone activity, ATP-binding subunit
SSU05_0506	3.565	collagenase-like protease
SSU05_0811	2.236	subtilisin-like serine protease
SSU05_1390	3.6395	SsrA-binding protein
SSU05_1884	2.4713	stomatin/prohibitin homolog
SSU05_1982	13.8094	subtilisin-like serine protease
SSU05_2082	3.1387	metalloendopeptidase
SSU05_0283	2.5742	ABC-type transport system involved in cytochrome bd biosynthesis, ATPase and permease component

downregulated

ID	FC	Description
SSU05_0015	2.4054	ATP-dependent Zn protease
SSU05_0237	2.8462	Thiol-disulfide isomerase and thioredoxin
SSU05_0328	20.5255	trigger factor
SSU05_0505	3.2754	collagenase-like protease

SSU05_0643	4.3546	glutathione S-transferase
SSU05_0645	8.0903	glutathione peroxidase
SSU05_0794	4.7749	pyrrolidone-carboxylate peptidase
SSU05_0837	3.8595	ATP-dependent protease ATP-binding subunit ClpX
SSU05_0864	4.1298	putative replication initiator protein
SSU05_1206	17.3328	NrdH-redoxin
SSU05_1329	4.108	glutathione S-transferase
SSU05_1344	9.5618	peptidyl-prolyl cis-trans isomerase
SSU05_1383	2.2323	thiol peroxidase
SSU05_1478	5.0108	Zn-dependent protease
SSU05_0140	15.0513	Thiol-disulfide isomerase and thioredoxin
SSU05_1550	3.8232	ATP-dependent Clp protease proteolytic subunit
SSU05_1551	19.8806	putative ATP-dependent Clp protease, proteolytic subunit
SSU05_1878	5.7022	adaptor protein

Replication, recombination and repair

upregulated

ID	FC	Description
SSU05_0054	2.5239	transposase
SSU05_0235	2.5937	mismatch repair ATPase
SSU05_0585	6.8078	transposase
SSU05_0587	11.6409	transposase
SSU05_0754	3.1542	ATP-dependent nuclease, subunit B
SSU05_0757	2.7534	Type IIA topoisomerase (DNA gyrase/topo II, topoisomerase IV), B subunit
SSU05_0810	2.284	transposase
SSU05_0979	3.5166	C-5 cytosine-specific DNA methylase
SSU05_1226	31.4541	transposase
SSU05_1424	2.0016	transposase
SSU05_2123	3.7	DNA mismatch repair protein MutS
SSU05_1567	4.3738	endonuclease

downregulated

ID	FC	Description
SSU05_0067	2.2984	Holliday junction resolvase-like protein
SSU05_0133	6.2981	adenine-specific DNA methylase
SSU05_0662	14.6054	DNA polymerase III subunit delta'
SSU05_0671	9.3897	exonuclease III
SSU05_0672	11.1805	putative 3'-exo-deoxyribonuclease
SSU05_0682	3.4641	hypothetical protein
SSU05_0685	2.8452	IS200 family transposase
SSU05_0749	11.0686	excinuclease ABC subunit C
SSU05_0813	43.9486	EndoIII-related endonuclease
SSU05_0917	14.3111	Tn916, transposase
SSU05_0996	5.5771	ribonuclease HII

SSU05_1052	2.5337	A/G-specific DNA glycosylase
SSU05_1071	2.7788	DNA repair protein
SSU05_1072	2.7849	DNA repair protein
SSU05_1073	3.2336	putative DNA repair protein
SSU05_1075	6.4925	DNA gyrase subunit A
SSU05_1090	5.7052	IS200 family transposase
SSU05_1305	2.488	putative primosome component and related proteins
SSU05_1429	2.9415	DNA primase
SSU05_1507	6.2863	transposase
SSU05_1529	5.037	prophage Lp3 protein 1, integrase
SSU05_1540	14.7335	DNA polymerase III subunit delta
SSU05_1627	2.3773	DNA polymerase III subunits gamma and tau
SSU05_1637	2.4323	IS200 family transposase
SSU05_1645	3.4491	transposase
SSU05_1646	2.8237	transposase
SSU05_1647	5.0737	nucleoid DNA-binding protein
SSU05_1651	2.0155	ATPase involved in DNA repair
SSU05_1681	14.7456	hypothetical protein
SSU05_1833	14.0341	single-stranded DNA-binding protein
SSU05_1863	2.2826	transposase
SSU05_1913	5.0178	transposase
SSU05_1954	4.59	DNA polymerase III PolC
SSU05_2010	2.6105	small primase-like protein
SSU05_2011	2.3594	Mg-dependent DNase
SSU05_2158	9.0472	replicative DNA helicase
SSU05_2159	6.3258	replicative DNA helicase
SSU05_2182	5.5638	recombination protein F
SSU05_0008	2.6416	transcription-repair coupling factor

Signal transduction mechanisms

upregulated

ID	FC	Description
SSU05_0348	4.8614	universal stress protein UspA-like nucleotide-binding protein
SSU05_0736	2.2868	hypothetical protein
SSU05_1686	3.3411	putative sensor histidine kinase
SSU05_1911	4.8605	sensor histidine kinase, putative
SSU05_2149	2.1608	two-component sensor histidine kinase
SSU05_1362	2.9685	glutamine ABC transporter substrate-binding protein

downregulated

ID	FC	Description
SSU05_0420	5.0778	S-ribosylhomocysteinase
SSU05_0468	2.7388	membrane GTPase involved in stress response
SSU05_0883	2.9924	Signal transduction histidine kinase
SSU05_0906	2.2252	NisK

SSU05_1358	6.2904	Signal transduction histidine kinase
SSU05_2161	4.1618	signaling protein
SSU05_2162	15.2917	signaling protein
SSU05_0552	2.9937	amino acid ABC transporter, amino acid-binding protein
SSU05_1017	13.5542	amino acid ABC transporter periplasmic protein
SSU05_1018	3.3965	putative amino acid transporter, amino acid-binding protein
SSU05_1026	3.0263	amino acid ABC transporter periplasmic protein
SSU05_1880	5.2855	hypothetical protein
SSU05_1878	5.7022	adaptor protein
SSU05_0884	2.5562	response regulator
SSU05_0885	2.8988	response regulator
SSU05_0907	2.1509	NisR
SSU05_1095	6.9893	response regulator

Secondary metabolites biosynthesis, transport and catabolism

upregulated

ID	FC	Description
SSU05_1056	2.0661	ABC-type multidrug transport system, ATPase and permease component
SSU05_1802	3.0731	3-oxoacyl-(acyl carrier protein) synthase II
SSU05_1156	67.2471	D-mannonate oxidoreductase
SSU05_1225	6.3536	gluconate 5-dehydrogenase
SSU05_1587	6.6311	3-ketoacyl-(acyl-carrier-protein) reductase
SSU05_1803	2.6812	3-ketoacyl-(acyl-carrier-protein) reductase
SSU05_1053	3.7826	hypothetical protein

downregulated

ID	FC	Description
SSU05_1806	2.1844	acyl carrier protein

Transcription

upregulated

ID	FC	Description
SSU05_1341	2.1276	transcriptional regulator
SSU05_1391	2.051	exoribonuclease R
SSU05_1559	2.3889	AraC-type DNA-binding domain-containing protein
SSU05_1573	4.5131	transcriptional regulator
SSU05_1590	6.7514	transcriptional regulator
SSU05_1612	13.3778	transcriptional accessory protein
SSU05_1745	40.4533	transcriptional regulator
SSU05_1819	9.8561	transcriptional regulator
SSU05_1933	4.5204	transcriptional regulator
SSU05_2066	8.4904	transcriptional regulator
SSU05_2076	18.3793	transcriptional antiterminator
SSU05_2087	2.4506	putative metal-dependent transcriptional regulator
SSU05_2137	6.3308	transcriptional regulator

SSU05_1816	2.5313	transcriptional regulator/sugar kinase
SSU05_1685	5.4842	response regulator
SSU05_0113	16.3797	transcriptional regulator
SSU05_0122	2.235	DNA-directed RNA polymerase subunit beta'
SSU05_0167	10.3394	transcriptional regulator
SSU05_0187	63.1387	transcriptional antiterminator
SSU05_0260	2.841	transcriptional regulator
SSU05_0264	12.3416	transcriptional regulator
SSU05_0298	33.784	heat-inducible transcription repressor
SSU05_0323	2.6183	hypothetical protein
SSU05_0324	2.7732	transcriptional regulator
SSU05_0359	6.0691	transcriptional regulator
SSU05_0447	7.2452	transcriptional regulator
SSU05_0528	16.1474	sigma24 homolog
SSU05_0541	2.8084	transcriptional regulator
SSU05_0608	3.2408	transcriptional regulator
SSU05_0916	4.237	putative Abi-alpha protein
SSU05_1051	3.4494	transcriptional regulator
SSU05_1131	3.8506	transcriptional regulator
SSU05_1232	80.0469	Cro/CI family transcriptional regulator
SSU05_0822	3.949	sugar metabolism transcriptional regulator
SSU05_0832	2.8729	transcriptional regulator/sugar kinase
SSU05_0562	3.1206	hypothetical protein
**downregulated**		
ID	FC	Description
SSU05_1491	3.4725	transcriptional antiterminator
SSU05_1527	19.3589	transcriptional regulator
SSU05_1716	2.9571	transcription elongation factor GreA
SSU05_1730	2.787	transcriptional regulator NrdR
SSU05_1820	9.7056	transcription termination factor
SSU05_1821	7.2512	transcription termination factor
SSU05_1858	3.3149	transcriptional regulator
SSU05_1976	3.9445	transcriptional regulator CtsR
SSU05_1983	7.0829	transcription antitermination protein NusG
SSU05_2039	15.4438	transcriptional regulator
SSU05_2056	6.2987	transcriptional antiterminator
SSU05_2168	4.0841	transcriptional regulatory protein
SSU05_1359	5.2371	response regulator
SSU05_1360	5.6606	response regulator
SSU05_2030	4.2778	response regulator
SSU05_2090	11.097	RevS
SSU05_0005	3.0077	transcriptional regulator
SSU05_0095	3.0518	DNA-directed RNA polymerase subunit alpha

SSU05_0232	6.5208	transcriptional regulator
SSU05_0350	3.5745	transcriptional repressor CodY
SSU05_0395	2.5203	transcriptional regulator
SSU05_0411	9.0205	cold shock protein
SSU05_0423	2.1887	DNA-directed RNA polymerase subunit omega
SSU05_0564	3.4657	Cps2A
SSU05_0655	8.1696	hypothetical protein
SSU05_0784	7.0963	CpsY
SSU05_0874	31.1106	transcriptional regulator
SSU05_0937	2.459	transcriptional regulator
SSU05_1012	40.2735	transcriptional regulator
SSU05_1136	7.6112	transcriptional regulator
SSU05_1162	7.3313	transcriptional regulator
SSU05_1191	3.4109	dsRNA-specific ribonuclease
SSU05_1210	6.7622	transcriptional regulator
SSU05_0706	4.8847	sugar metabolism transcriptional regulator
SSU05_1045	11.349	sugar metabolism transcriptional regulator
SSU05_0008	2.6416	transcription-repair coupling factor
SSU05_0884	2.5562	response regulator
SSU05_0885	2.8988	response regulator
SSU05_0907	2.1509	NisR
SSU05_1095	6.9893	response regulator
SSU05_1574	13.3328	superfamily II DNA/RNA helicase
**Translation**		
**upregulated**		
ID	FC	Description
SSU05_0278	3.1439	histidyl-tRNA synthetase
SSU05_0304	2.5582	amidase
SSU05_0368	2.0716	RNA-binding protein
SSU05_0388	11.5129	N-formylmethionyl-tRNA deformylase
SSU05_0439	10.1464	ribosome-associated protein Y (PSrp-1)
SSU05_0459	7.4875	valyl-tRNA synthetase
SSU05_0489	2.2833	isoleucyl-tRNA synthetase
SSU05_0603	2.096	SAM-dependent methyltransferase related to tRNA (uracil-5-)-methyltransferase
SSU05_0619	3.2309	YjgF family translation initiation inhibitor
SSU05_1236	2.8518	alanyl-tRNA synthetase
SSU05_1245	2.0348	methionyl-tRNA synthetase
SSU05_1366	3.6648	threonyl-tRNA synthetase
SSU05_1764	3.9854	glycyl-tRNA synthetase subunit beta
SSU05_1765	2.4084	glycyl-tRNA synthetase subunit alpha
**downregulated**		
ID	FC	Description

SSU05_0009	2.1704	S4 paralog
SSU05_0070	8.1542	ribosomal protein S10
SSU05_0071	6.9948	50S ribosomal protein L3
SSU05_0072	2.469	50S ribosomal protein L4
SSU05_0073	2.2679	50S ribosomal protein L23
SSU05_0089	2.2101	ribosomal protein L15
SSU05_0092	2.5319	translation initiation factor IF-1
SSU05_0093	3.8527	30S ribosomal protein S13
SSU05_0094	2.79	30S ribosomal protein S11
SSU05_0096	2.2818	ribosomal protein L17
SSU05_0097	2.3311	ribosomal protein L17
SSU05_0150	3.5194	30S ribosomal protein S12
SSU05_0151	3.2605	30S ribosomal protein S7
SSU05_0277	3.3083	50S ribosomal protein L32
SSU05_0445	4.3449	hypothetical protein
SSU05_0530	4.8825	Streptococcus oralis sp|P33170|EFTU_STROR Elongation factor Tu (EF-Tu)
SSU05_0769	182.7311	putative ribosomal protein S1-like DNA-binding protein
SSU05_0770	22.0876	hypothetical protein
SSU05_0772	23.4022	putative ribosomal protein S1-like DNA-binding protein
SSU05_0781	11.4288	50S ribosomal protein L21
SSU05_0782	3.4432	50S ribosomal protein L27
SSU05_0786	12.7974	YlyB
SSU05_0796	6.1299	30S ribosomal protein S16
SSU05_0819	11.652	16S rRNA-processing protein RimM
SSU05_0820	14.7769	tRNA (guanine-N(1)-)-methyltransferase
SSU05_0829	6.0209	tRNA CCA-pyrophosphorylase
SSU05_0922	3.2826	translation elongation factor (GTPases)
SSU05_0983	3.5218	50S ribosomal protein L7/L12
SSU05_0984	9.1043	ribosomal protein L10
SSU05_1058	6.8998	pseudouridylate synthase, 23S RNA-specific
SSU05_1114	5.844	pseudouridine synthase
SSU05_1115	20.1716	pseudouridine synthase
SSU05_1152	3.9421	phenylalanyl-tRNA synthetase subunit alpha
SSU05_1270	2.6769	translation initiation factor IF-3
SSU05_1316	2.5359	tRNA delta(2)-isopentenylpyrophosphate transferase
SSU05_1331	8.8599	50S ribosomal protein L1
SSU05_1332	10.617	50S ribosomal protein L11
SSU05_1374	5.0337	queuine tRNA-ribosyltransferase
SSU05_1412	2.7423	peptide chain release factor 2
SSU05_1433	4.5712	30S ribosomal protein S21
SSU05_1531	4.5065	50S ribosomal protein L31 type B
SSU05_1535	7.638	30S ribosomal protein S14
SSU05_1618	4.4271	acetyltransferase

SSU05_1697	6.8004	rRNA methyltransferase
SSU05_1698	8.3645	hypothetical protein
SSU05_1713	2.5505	rRNA methylase
SSU05_1823	5.3943	translation initiation factor 5A (eIF-5A)
SSU05_1824	4.4488	putative translation elongation factor EF-P
SSU05_1832	18.0651	30S ribosomal protein S18
SSU05_1834	14.2551	ribosomal protein S6
SSU05_1859	5.1465	histone acetyltransferase HPA2-like acetyltransferase
SSU05_1897	3.0578	30S ribosomal protein S9
SSU05_1898	5.0932	50S ribosomal protein L13
SSU05_1924	2.4996	cysteinyl-tRNA synthetase
SSU05_1934	4.0683	30S ribosomal protein S15
SSU05_1940	2.9236	16S rRNA uridine-516 pseudouridylate synthase family protein
SSU05_1944	17.7049	peptide deformylase
SSU05_1979	2.8738	elongation factor Ts
SSU05_1980	8.243	30S ribosomal protein S2
SSU05_2001	2.052	dimethyladenosine transferase
SSU05_2015	63.5159	ribonuclease P
SSU05_2156	4.3361	30S ribosomal protein S4
SSU05_2160	6.6388	50S ribosomal protein L9
SSU05_2184	13.605	tryptophanyl-tRNA synthetase II
SSU05_1574	13.3328	superfamily II DNA/RNA helicase
SSU05_0344	4.0129	L-asparaginase/ Glu-tRNAGln amidotransferase subunit D
SSU05_0345	2.7549	L-asparaginase/ Glu-tRNAGln amidotransferase subunit D
SSU05_1500	5.1633	cytotoxic translational repressor of toxin-antitoxin stability system
SSU05_0886	4.2275	cytotoxic translational repressor of toxin-antitoxin stability system

General function prediction only

upregulated

ID	FC	Description
SSU05_0183	2.0877	flavoprotein
SSU05_0255	26.4034	HAD superfamily hydrolase
SSU05_0256	6.4202	ABC-type uncharacterized transport system, permease component
SSU05_0257	4.1398	ABC-type uncharacterized transport system, permease component
SSU05_0258	3.998	ABC-type uncharacterized transport system, ATPase component
SSU05_0279	2.5925	alcohol dehydrogenase
SSU05_0320	2.1234	alpha/beta superfamily hydrolase/acyltransferase
SSU05_0321	3.9544	dehydrogenase
SSU05_0365	2.8311	HAD superfamily hydrolase
SSU05_0366	3.3017	GTPase

SSU05_0367	3.0147	GTPase
SSU05_0378	3.3906	nucleotidyltransferase
SSU05_0379	2.6165	nucleotidyltransferase
SSU05_0383	10.0497	putative ATP-binding protein
SSU05_0457	2.4356	lactoylglutathione lyase and related lyases
SSU05_0532	3.9342	hypothetical protein
SSU05_0533	2.0757	HD superfamily phosphohydrolase
SSU05_0620	4.0486	hypothetical protein
SSU05_0625	58.1332	histone acetyltransferase HPA2-like acetyltransferase
SSU05_0902	2.6303	HAD superfamily hydrolase
SSU05_0968	2.0254	Tn5252, Orf28
SSU05_0992	19.4796	NAD(FAD)-dependent dehydrogenase
SSU05_0994	2.298	hemolysin III homolog
SSU05_1127	3.5173	HAD superfamily hydrolase
SSU05_1155	60.3204	phosphatase
SSU05_1253	4.3291	ABC-type uncharacterized transport system, ATPase component
SSU05_1254	6.9112	ABC-type uncharacterized transport system, permease component
SSU05_1255	6.3546	ABC-type uncharacterized transport system, permease component
SSU05_1256	6.3158	hypothetical protein
SSU05_1257	7.1673	ABC transporter permease protein
SSU05_1313	2.4449	ribonuclease Z
SSU05_1459	5.1292	beta-propeller domain-containing protein
SSU05_1484	3.863	ABC-type uncharacterized transport system, ATPase component
SSU05_1485	4.2683	ABC-type uncharacterized transport system, ATPase component
SSU05_1486	3.6896	ABC-type uncharacterized transport system, permease component
SSU05_1487	2.9356	ABC-type uncharacterized transport system, periplasmic component
SSU05_1566	5.2183	O-methyltransferase
SSU05_1932	17.3782	glucokinase regulatory protein
SSU05_1968	4.0377	DNA nuclease
**downregulated**		
ID	FC	Description
SSU05_0228	3.231	dehydrogenase and related proteins
SSU05_0234	2.9073	hypothetical protein
SSU05_0265	9.3433	putative effector of murein hydrolase LrgA
SSU05_0346	3.5722	HAD superfamily hydrolase
SSU05_0362	6.7465	DMT family permease

SSU05_0406	3.0964	hypothetical protein
SSU05_0415	6.3263	Holliday junction-specific endonuclease
SSU05_0421	6.538	hypothetical protein
SSU05_0482	3.5329	TIM-barrel fold family protein
SSU05_0555	8.5185	Zn-dependent hydrolase, including glyoxylases
SSU05_0664	11.2621	methyltransferase
SSU05_0730	2.3841	NAD(FAD)-dependent dehydrogenase
SSU05_0751	2.1991	GTPase ObgE
SSU05_0797	5.7203	RNA-binding protein
SSU05_0838	3.8543	GTPase
SSU05_0851	2.7411	hypothetical protein
SSU05_0855	15.8487	Short-chain alcohol dehydrogenase of unknown specificity
SSU05_0989	23.7066	acetyltransferase
SSU05_0997	5.8179	ribosomal biogenesis GTPase
SSU05_0998	4.0151	Fe-S-cluster oxidoreductase
SSU05_1069	2.6729	redox-sensing transcriptional repressor Rex
SSU05_1070	9.3735	hypothetical protein
SSU05_1083	2.7221	ABC transporter periplasmic protein
SSU05_1130	5.056	permease
SSU05_1182	6.7124	permease
SSU05_1239	4.0228	O-methyltransferase
SSU05_1264	11.1171	SAM-dependent methyltransferase
SSU05_1304	2.6271	SAM-dependent methyltransferase
SSU05_1328	4.5311	O-antigen and teichoic acid export protein
SSU05_1357	6.4008	beta-lactamase superfamily hydrolase
SSU05_1399	2.1567	putative metalloprotease
SSU05_1427	9.3097	metal-sulfur cluster biosynthetic protein
SSU05_1454	2.1099	tRNA modification GTPase TrmE
SSU05_1479	5.3686	esterase
SSU05_1496	2.1141	permease
SSU05_1499	2.4847	HAD superfamily hydrolase
SSU05_1613	2.1319	aldo/keto reductase family oxidoreductase
SSU05_1644	8.4038	histone acetyltransferase HPA2-like acetyltransferase
SSU05_1668	24.0532	hemolysin-like protein
SSU05_1676	5.3431	Fe-S-cluster redox protein
SSU05_1677	4.8084	Fe-S-cluster redox protein
SSU05_1710	8.6086	putative integral membrane protein
SSU05_1711	11.1116	hypothetical protein
SSU05_1717	6.5867	periplasmic solute-binding protein
SSU05_1731	4.0119	permease
SSU05_1766	10.6685	Phage envelope protein
SSU05_1773	2.4165	glutamine amidotransferase, class I
SSU05_1777	33.4395	HAD superfamily hydrolase
SSU05_1810	4.7431	phosphatase/phosphohexomutase

SSU05_1851	2.7529	tRNA (guanine-N(7)-)-methyltransferase
SSU05_1860	9.4968	ATPase or kinase
SSU05_1861	3.0355	kinase related to dihydroxyacetone kinase
SSU05_1894	2.444	metal-sulfur cluster biosynthetic protein
SSU05_1896	2.3774	hypothetical protein
SSU05_1928	2.2833	hypothetical protein
SSU05_1989	3.9742	aminoglycoside phosphotransferase
SSU05_1994	2.3118	ribosome-associated GTPase
SSU05_2007	4.0362	hypothetical protein
SSU05_2013	5.9016	RNA-binding protein
SSU05_2020	7.4977	small molecule binding protein
SSU05_2070	2.6707	surface antigen
SSU05_2089	2.6088	hypothetical protein
SSU05_2179	5.7556	Zn-dependent peptidase
SSU05_2180	34.4709	Zn-dependent peptidase

others

upregulated

ID	FC	Description
SSU05_0114	12.4331	hypothetical protein
SSU05_0136	2.3975	hypothetical protein
SSU05_0144	8.1238	hypothetical protein
SSU05_0162	2.0067	hypothetical protein
SSU05_0173	41.4474	hypothetical protein
SSU05_0180	10.405	hypothetical protein
SSU05_0182	2.8352	hypothetical protein
SSU05_0189	75.9693	ascorbate-specific PTS system enzyme IIC
SSU05_0213	18.5698	hypothetical protein
SSU05_0242	2.801	transcriptional regulator PlcR, putative
SSU05_0244	3.9685	hypothetical protein
SSU05_0245	5.414	hypothetical protein
SSU05_0246	5.3471	hypothetical protein
SSU05_0247	4.2546	hypothetical protein
SSU05_0261	6.6685	hypothetical protein
SSU05_0262	12.8673	hypothetical protein
SSU05_0263	15.2572	hypothetical protein
SSU05_0272	2.3988	translation initiation factor 2 GTPase
SSU05_0274	8.3211	methyl-accepting chemotaxis protein
SSU05_0282	2.7289	hypothetical protein
SSU05_0296	4.7558	hypothetical protein
SSU05_0301	19.1171	hypothetical protein
SSU05_0308	22.7302	hypothetical protein
SSU05_0313	2.407	hypothetical protein
SSU05_0371	5.614	histone acetyltransferase HPA2-like acetyltransferase
SSU05_0381	2.0827	hypothetical protein

SSU05_0382	7.556	hypothetical protein
SSU05_0384	10.2142	hypothetical protein
SSU05_0385	10.0099	hypothetical protein
SSU05_0386	14.3961	hypothetical protein
SSU05_0396	2.8196	metal-dependent hydrolase
SSU05_0397	5.7399	phosphotransferase system IIC component, glucose/maltose/N-acetylglucosamine-specific
SSU05_0405	4.164	hypothetical protein
SSU05_0458	6.7809	shikimate kinase
SSU05_0460	3.0994	hypothetical protein
SSU05_0461	2.7823	hypothetical protein
SSU05_0466	2.4094	hypothetical protein
SSU05_0473	2.4427	ribonucleases G and E
SSU05_0523	4.6848	hypothetical protein
SSU05_0529	23.1604	hypothetical protein
SSU05_0553	2.2323	hypothetical protein
SSU05_0561	2.3681	hypothetical protein
SSU05_0586	13.5419	hypothetical protein
SSU05_0588	4.3968	transposase
SSU05_0590	5.0194	hypothetical protein
SSU05_0594	2.5636	ATPase involved in DNA repair
SSU05_0604	2.8398	hypothetical protein
SSU05_0612	3.954	hypothetical protein
SSU05_0615	4.8451	hypothetical protein
SSU05_0617	4.1514	hypothetical protein
SSU05_0621	2.846	hypothetical protein
SSU05_0622	2.6899	hypothetical protein
SSU05_0628	616.5981	hypothetical protein
SSU05_0637	2.7228	hypothetical protein
SSU05_0659	2.5367	hypothetical protein
SSU05_0673	2.832	hypothetical protein
SSU05_0674	4.9002	hypothetical protein
SSU05_0676	5.869	integral membrane protein
SSU05_0679	2.6564	hypothetical protein
SSU05_0739	7.6847	hypothetical protein
SSU05_0747	5.4176	permease
SSU05_0766	2.5087	branched-chain amino acid aminotransferase/4-amino-4-deoxychorismate lyase
SSU05_0812	2.6014	subtilisin-like serine protease
SSU05_0845	5.9368	hypothetical protein
SSU05_0888	3.9846	peptide ABC transporter permease
SSU05_0889	2.7054	peptide ABC transporter permease
SSU05_0890	3.2492	peptide ABC transporter permease
SSU05_0940	4.2478	hypothetical protein

SSU05_0941	2.8046	DNA primase (type)
SSU05_0952	3.2388	recombinase
SSU05_0970	2.2396	hypothetical protein
SSU05_0971	3.1257	ABC-type cobalt transport system, permease component CbiQ and related transporters
SSU05_0972	2.2999	hypothetical protein
SSU05_0975	2.292	protease, putative
SSU05_0976	2.9698	hypothetical protein
SSU05_0978	3.144	hypothetical protein
SSU05_0981	2.608	hypothetical protein
SSU05_1005	2.7597	hypothetical protein
SSU05_1049	32.1277	hypothetical protein
SSU05_1050	81.2589	hypothetical protein
SSU05_1055	2.492	ABC-type multidrug transport system, ATPase and permease component
SSU05_1092	4.4578	hypothetical protein
SSU05_1116	2.9871	hypothetical protein
SSU05_1118	3.3866	hypothetical protein
SSU05_1125	4.9671	hypothetical protein
SSU05_1126	5.5303	hypothetical protein
SSU05_1132	15.664	hypothetical protein
SSU05_1133	17.8455	hypothetical protein
SSU05_1134	11.3151	hypothetical protein
SSU05_1193	4.5349	hypothetical protein
SSU05_1211	37.6074	hypothetical protein
SSU05_1212	358.8436	hyaluronidase
SSU05_1213	158.7394	hyaluronidase
SSU05_1214	167.346	hyaluronidase
SSU05_1215	158.1628	hyaluronidase
SSU05_1220	46.7767	hypothetical protein
SSU05_1227	56.2443	metal-dependent membrane protease
SSU05_1228	52.6939	hypothetical protein
SSU05_1229	50.5674	hypothetical protein
SSU05_1230	94.7426	hypothetical protein
SSU05_1231	87.448	hypothetical protein
SSU05_1233	77.5884	surface antigen negative regulator Par
SSU05_1311	17.8593	hypothetical protein
SSU05_1371	3.1106	ribonucleases G and E
SSU05_1422	2.4167	hypothetical protein
SSU05_1425	3.2418	transposase
SSU05_1456	3.149	hypothetical protein
SSU05_1457	5.9847	hypothetical protein
SSU05_1458	4.9608	hypothetical protein
SSU05_1476	2.0642	ABC transporter ATPase

SSU05_1482	2.7537	hypothetical protein
SSU05_1498	3.9777	hypothetical protein
SSU05_1563	12.6113	hypothetical protein
SSU05_1564	8.884	hypothetical protein
SSU05_1565	7.7499	hypothetical protein
SSU05_1568	2.9542	hypothetical protein
SSU05_1572	4.9713	integral membrane protein
SSU05_1578	10.8939	hypothetical protein
SSU05_1585	5.7359	hypothetical protein
SSU05_1586	4.3994	hypothetical protein
SSU05_1588	13.5656	hypothetical protein
SSU05_1589	13.687	hypothetical protein
SSU05_1591	6.2676	hypothetical protein
SSU05_1593	2.763	ABC transporter membrane-spanning permease - unknown substrate
SSU05_1611	16.2258	hypothetical protein
SSU05_1615	27.9185	hypothetical protein
SSU05_1725	3.8362	major facilitator superfamily permease
SSU05_1746	44.5543	hemolysin-like protein
SSU05_1747	105.7446	hypothetical protein
SSU05_1748	69.6212	hypothetical protein
SSU05_1749	82.1157	major facilitator superfamily permease
SSU05_1750	74.2755	hypothetical protein
SSU05_1751	27.8041	hypothetical protein
SSU05_1754	3.0702	major membrane immunogen, membrane-anchored lipoprotein
SSU05_1762	8.4133	hypothetical protein
SSU05_1763	2.1024	hypothetical protein
SSU05_1788	6.9604	hypothetical protein
SSU05_1789	5.286	hypothetical protein
SSU05_1843	2.3026	dockerin type I
SSU05_1857	4.3992	methyl-accepting chemotaxis protein
SSU05_1872	2.274	hypothetical protein
SSU05_1900	2.3787	hypothetical protein
SSU05_1903	2.1256	hypothetical protein
SSU05_1920	10.7053	hypothetical protein
SSU05_1929	17.4591	hypothetical protein
SSU05_1930	11.5361	outer surface protein
SSU05_1959	77.7527	hypothetical protein
SSU05_2021	2.7323	hypothetical protein
SSU05_2072	29.3216	hypothetical protein
SSU05_2077	7.6731	hypothetical protein
SSU05_2091	3.7303	hypothetical protein
SSU05_2101	2.1386	hypothetical protein
SSU05_2106	2.4801	16S ribosomal RNA methyltransferase RsmE

SSU05_2107	2.6522	ribosomal protein L11 methylase
SSU05_2120	2.5631	FOG: Transposase and inactivated derivative
SSU05_2136	4.2734	integral membrane protein
SSU05_2194	2.6947	transcriptional regulator
**downregulated**		
ID	FC	Description
SSU05_0004	4.4223	hypothetical protein
SSU05_0011	2.7223	hypothetical protein
SSU05_0020	2.0746	hypothetical protein
SSU05_0040	5.6027	hypothetical protein
SSU05_0053	17.5879	transcriptional regulator
SSU05_0069	5.7782	hypothetical protein
SSU05_0123	3.7449	hypothetical protein
SSU05_0134	5.5224	adenine-specific DNA methylase
SSU05_0139	11.454	hypothetical protein
SSU05_0158	6.1733	hypothetical protein
SSU05_0194	7.1875	hypothetical protein
SSU05_0227	3.5429	dehydrogenase and related proteins
SSU05_0229	7.0528	FOG: LysM repeat
SSU05_0238	2.2506	hypothetical protein
SSU05_0251	2.0879	ATPase
SSU05_0273	4.1016	methyl-accepting chemotaxis protein
SSU05_0400	5.4988	hypothetical protein
SSU05_0402	10.1647	hypothetical protein
SSU05_0403	4.5583	hypothetical protein
SSU05_0404	2.3403	hypothetical protein
SSU05_0484	2.862	hypothetical protein
SSU05_0485	3.3234	integral membrane protein
SSU05_0486	3.5854	S4-like domain-containing protein
SSU05_0504	5.9227	hypothetical protein
SSU05_0521	2.4583	hypothetical protein
SSU05_0522	22.9416	hypothetical protein
SSU05_0571	2.3388	Cps2H
SSU05_0572	2.3485	Cps2I
SSU05_0633	10.7088	hypothetical protein
SSU05_0635	8.0125	hypothetical protein
SSU05_0642	3.6342	putative low temperature requirement A protein
SSU05_0657	56.0271	hypothetical protein
SSU05_0660	8.0325	hypothetical protein
SSU05_0663	7.2602	DNA replication intiation control protein YabA
SSU05_0677	3.4683	integral membrane protein
SSU05_0680	12.9182	hypothetical protein
SSU05_0687	3.5098	hypothetical protein

SSU05_0698	2.2387	hypothetical protein
SSU05_0750	64.1982	hypothetical protein
SSU05_0752	4.6551	hypothetical protein
SSU05_0756	15.339	putative glycerol-3-phosphate acyltransferase PlsY
SSU05_0773	35.3856	putative ribosomal protein S1-like DNA-binding protein
SSU05_0779	5.2896	branched-chain amino acid permease
SSU05_0783	2.2814	C-P lyase regulatory protein
SSU05_0787	12.5071	hypothetical protein
SSU05_0788	8.3699	hypothetical protein
SSU05_0795	3.7131	hypothetical protein
SSU05_0826	7.374	hypothetical protein
SSU05_0827	11.5629	hypothetical protein
SSU05_0836	20.9009	hypothetical protein
SSU05_0840	4.3932	hypothetical protein
SSU05_0853	6.2103	hypothetical protein
SSU05_0854	3.8907	lytic murein transglycosylase
SSU05_0856	19.8157	translation initiation factor 1
SSU05_0857	20.3704	hypothetical protein
SSU05_0863	9.838	hypothetical protein
SSU05_0865	2.3629	hypothetical protein
SSU05_0866	7.3239	hypothetical protein
SSU05_0867	4.2704	hypothetical protein
SSU05_0869	3.8852	hypothetical protein
SSU05_0870	6.5012	hypothetical protein
SSU05_0871	36.5667	hypothetical protein
SSU05_0875	26.8925	putative DNA-binding protein
SSU05_0877	2.9965	hypothetical protein
SSU05_0887	3.7834	hypothetical protein
SSU05_0912	5.4691	putative asparagine synthetase
SSU05_0914	3.722	hypothetical protein
SSU05_0915	2.624	hypothetical protein
SSU05_0936	2.2063	Signal recognition particle GTPase
SSU05_0995	9.2615	hypothetical protein
SSU05_1019	3.3574	hypothetical protein
SSU05_1048	6.4025	integrase
SSU05_1060	59.258	hypothetical protein
SSU05_1061	11.1901	hypothetical protein
SSU05_1062	8.6538	hypothetical protein
SSU05_1067	13.1678	hypothetical protein
SSU05_1068	13.5116	hypothetical protein
SSU05_1097	3.9423	hypothetical protein
SSU05_1108	2.239	hypothetical protein
SSU05_1110	22.6542	hypothetical protein
SSU05_1112	2.8773	Kef-type K+ transporter NAD-binding component

SSU05_1129	7.5915	hemolysin III homolog
SSU05_1135	40.0862	hypothetical protein
SSU05_1137	4.625	hypothetical protein
SSU05_1146	3.3325	hypothetical protein
SSU05_1148	7.6774	hypothetical protein
SSU05_1161	76.6499	transcriptional regulator
SSU05_1168	7.29	hypothetical protein
SSU05_1169	8.8486	hypothetical protein
SSU05_1181	2.1809	hypothetical protein
SSU05_1183	2.0428	hypothetical protein
SSU05_1238	5.3348	parvulin-like peptidyl-prolyl isomerase
SSU05_1242	2.0242	hypothetical protein
SSU05_1247	10.3159	hypothetical protein
SSU05_1265	17.6788	phosphoglycerol transferase/alkaline phosphatase superfamily protein
SSU05_1267	3.4661	Type IIA topoisomerase (DNA gyrase/topo II, topoisomerase IV), A subunit
SSU05_1276	5.2437	hypothetical protein
SSU05_1282	2.3892	hypothetical protein
SSU05_1283	2.675	hypothetical protein
SSU05_1289	4.7964	glycosyltransferase
SSU05_1291	11.7297	glycosyltransferase
SSU05_1299	2.4934	hypothetical protein
SSU05_1303	2.9904	hypothetical protein
SSU05_1317	5.6775	hypothetical protein
SSU05_1322	2.2434	hypothetical protein
SSU05_1325	2.3184	hypothetical protein
SSU05_1330	8.6707	hypothetical protein
SSU05_1342	4.9928	hypothetical protein
SSU05_1351	4.314	hypothetical protein
SSU05_1375	7.8627	integral membrane protein
SSU05_1413	2.4363	hypothetical protein
SSU05_1415	3.9644	glycopeptide antibiotics resistance protein
SSU05_1416	2.9653	hypothetical protein
SSU05_1420	8.5901	hypothetical protein
SSU05_1434	2.3937	hypothetical protein
SSU05_1469	2.3065	hypothetical protein
SSU05_1470	8.7756	hypothetical protein
SSU05_1501	6.2473	hypothetical protein
SSU05_1528	21.8172	Gp21 protein
SSU05_1552	5.4092	hypothetical protein
SSU05_1599	3.2441	hypothetical protein
SSU05_1610	3.3728	hypothetical protein
SSU05_1626	5.2472	ABC-type multidrug transport system, ATPase and permease

		component
SSU05_1639	153.2371	hypothetical protein
SSU05_1648	6.3539	hypothetical protein
SSU05_1650	2.9885	hypothetical protein
SSU05_1662	5.4606	hypothetical protein
SSU05_1672	3.6361	nicotinate phosphoribosyltransferase
SSU05_1675	9.0166	hypothetical protein
SSU05_1678	7.1023	hypothetical protein
SSU05_1684	5.6828	hypothetical protein
SSU05_1690	4.9766	similar to MF3 gene in Streptococcus pyogenes MGAS10394
SSU05_1696	6.9766	hypothetical protein
SSU05_1715	13.7257	preprotein translocase subunit YidC
SSU05_1718	7.5291	periplasmic solute-binding protein
SSU05_1758	5.6362	hypothetical protein
SSU05_1822	8.1873	hypothetical protein
SSU05_1853	6.1955	hypothetical protein
SSU05_1862	5.7194	hypothetical protein
SSU05_1869	2.1595	hypothetical protein
SSU05_1895	2.3596	hypothetical protein
SSU05_1923	3.1415	hypothetical protein
SSU05_1939	7.6534	hypothetical protein
SSU05_1953	5.3454	hypothetical protein
SSU05_1977	3.8447	putative bacterocin transport accessory protein, Bta
SSU05_1997	3.1544	transcriptional regulator
SSU05_2000	15.1777	major facilitator superfamily permease
SSU05_2002	4.8941	hypothetical protein
SSU05_2003	4.8255	hypothetical protein
SSU05_2008	2.6915	hypothetical protein
SSU05_2009	3.5846	hypothetical protein
SSU05_2012	5.2271	hypothetical protein
SSU05_2019	8.3247	hypothetical protein
SSU05_2031	4.7073	hypothetical protein
SSU05_2045	31.0855	hypothetical protein
SSU05_2050	4.5555	Serine/threonine protein phosphatase
SSU05_2110	3.3339	hypothetical protein
SSU05_2111	3.4538	hypothetical protein
SSU05_2112	4.9749	hypothetical protein
SSU05_2155	7.9354	Thiol-disulfide isomerase and thioredoxin
SSU05_2164	2.7282	NTP pyrophosphohydrolase including oxidative damage repair enzymes
SSU05_2178	3.6259	hypothetical protein
SSU05_2181	4.9566	hypothetical protein

Table S4. The regulated genes in Δ*relA* during glucose starvation

Amino acid transport and metabolism		
**Upregulated**		
ID	FC	Description
SSU05_0467	2.2512	asparagine synthetase AsnA
SSU05_0557	4.7342	gamma-glutamyl kinase
SSU05_0559	6.1613	pyrroline-5-carboxylate reductase
SSU05_0624	36.9506	arginine deiminase
SSU05_0626	37.5521	ornithine carbamoyltransferase
SSU05_0627	305.1105	carbamate kinase
SSU05_0675	4.3049	amino acid transporter
SSU05_0725	2.9638	glyoxalase family protein
SSU05_1030	3.4033	histone acetyltransferase HPA2-like acetyltransferase
SSU05_1387	10.088	amylase-binding protein B
SSU05_1548	3.1329	hypothetical protein
SSU05_1882	2.002	amino acid ABC transporter ATP-binding protein
SSU05_0021	3.7477	phosphoribosylpyrophosphate synthetase
SSU05_0719	4.6203	dihydrodipicolinate synthase
SSU05_0305	2.1106	amino acid ABC transporter periplasmic protein
SSU05_0552	3.6411	amino acid ABC transporter, amino acid-binding protein
**downregulated**		
ID	FC	Description
SSU05_0160	2.0742	glutamine synthetase
SSU05_0174	2.4336	putative integral membrane protein; branched-chain amino acid permease
SSU05_0435	17.3968	O-acetylserine lyase
SSU05_0494	2.8907	ABC-type amino acid transport system, permease component
SSU05_0496	2.2332	putative amino acid ABC transporter, ATP-binding protein
SSU05_0550	3.7518	amino acid (glutamine) ABC transporter, permease protein
SSU05_0596	3.0835	5-enolpyruvylshikimate-3-phosphate synthase
SSU05_0774	3.2174	threonine aldolase
SSU05_1361	2.0018	ABC-type polar amino acid transport system, ATPase component
SSU05_1536	4.0938	chorismate mutase
SSU05_1561	2.1578	bifunctional beta-cystathionase/maltose regulon repressor
SSU05_1598	2.1219	transaminase
SSU05_1649	2.0236	lysophospholipase L1 and related esterases
SSU05_1708	2.2683	putative diaminopimelate decarboxylase
SSU05_1709	2.1152	cysteine aminopeptidase C
SSU05_1825	3.059	aminopeptidase P; XAA-pro aminopeptidase
SSU05_2068	17.1823	ABC-type amino acid transport system, permease component
SSU05_0791	3.7124	carbamoyl phosphate synthase small subunit
SSU05_1065	3.1158	ribose-phosphate pyrophosphokinase

SSU05_1140	2.741	D-beta-hydroxybutyrate permease
SSU05_1017	11.8352	amino acid ABC transporter periplasmic protein
SSU05_1018	43.1707	putative amino acid transporter, amino acid-binding protein
SSU05_2069	49.8037	amino acid ABC transporter periplasmic protein
SSU05_0562	2.7625	hypothetical protein

Carbohydrate transport and metabolism

upregulated

ID	FC	Description
SSU05_0168	14.8776	ABC-type sugar transport system, periplasmic component
SSU05_0169	10.5862	ABC-type sugar transport system, periplasmic component
SSU05_0170	4.8345	ABC-type sugar transport system, permease component
SSU05_0171	4.6335	ABC-type sugar transport system, permease component
SSU05_0172	5.228	Alpha-galactosidase
SSU05_0205	4.3221	fructose-1-phosphate kinase-like protein
SSU05_0206	4.5297	fructose-1-phosphate kinase-like protein
SSU05_0219	2.6785	6-phosphogluconolactonase/glucosamine-6-phosphate isomerase/deaminase
SSU05_0230	3.4061	glycosidase
SSU05_0361	6.8379	galactose-1-phosphate uridylyltransferase
SSU05_0449	24.4761	Beta-galactosidase
SSU05_0450	15.6949	phosphotransferase system, mannose/fructose/N-acetylgalactosamine-specific component IIB
SSU05_0451	15.9021	phosphotransferase system, mannose/fructose/N-acetylgalactosamine-specific component IIC
SSU05_0452	18.1041	phosphotransferase system, mannose/fructose/N-acetylgalactosamine-specific component IID
SSU05_0634	2.2101	6-phosphogluconolactonase/glucosamine-6-phosphate isomerase/deaminase
SSU05_0686	2.0639	phosphomannomutase
SSU05_0823	9.8742	fructose-1-phosphate kinase-like protein
SSU05_0824	8.1463	phosphotransferase system, fructose-specific IIC component
SSU05_0825	10.6192	phosphotransferase system, fructose-specific IIC component
SSU05_0882	2.186	phosphomannomutase
SSU05_1037	2.1995	phosphotransferase system cellobiose-specific component IIC
SSU05_1038	5.3884	phosphotransferase system cellobiose-specific component IIA
SSU05_1040	2.7299	tagatose 1,6-diphosphate aldolase
SSU05_1153	8.4684	Beta-glucosidase-related glycosidase

SSU05_1154	12.0118	hypothetical protein
SSU05_1157	21.5499	mannonate dehydratase
SSU05_1158	23.8699	glucuronate isomerase
SSU05_1159	23.937	glucuronate isomerase
SSU05_1217	2.8577	phosphotransferase system, mannose/fructose/N-acetylgalactosamine-specific component IID
SSU05_1258	2.2677	tagatose 1,6-diphosphate aldolase
SSU05_1338	4.1065	ABC-type sugar transport system, periplasmic component
SSU05_1339	4.605	ABC-type sugar transport system, permease component
SSU05_1340	6.0569	ABC-type polysaccharide transport system, permease component
SSU05_1402	3.4905	N-acetylmannosamine-6-phosphate 2-epimerase
SSU05_1448	3.7894	phosphopentomutase
SSU05_1778	9.9961	putative PTS system, mannose-specific component IIAB
SSU05_1779	10.4141	mannose-specific PTS IIC
SSU05_1780	6.8207	mannose-specific PTS IID
SSU05_1817	9.6364	phosphotransferase system IIC component, glucose/maltose/N-acetylglucosamine-specific
SSU05_1818	3.3856	Beta-fructosidases (levanase/invertase)
SSU05_1907	5.2054	ABC-type sugar transport system, ATPase component
SSU05_1921	2.7647	putative alpha-1,2-mannosidase
SSU05_1957	79.7524	dihydroxyacetone kinase
SSU05_1958	70.9398	dihydroxyacetone kinase
SSU05_1960	43.9078	glycerol uptake facilitator and related permease (major Intrinsic protein family)
SSU05_2051	2.4994	glucose-6-phosphate isomerase
SSU05_2131	4.8018	4-alpha-glucanotransferase
SSU05_2132	6.5907	4-alpha-glucanotransferase
SSU05_0822	18.7725	sugar metabolism transcriptional regulator
SSU05_1045	4.194	sugar metabolism transcriptional regulator
SSU05_1259	2.2231	N-acetylglucosamine-6-phosphate deacetylase
**downregulated**		
ID	FC	Description
SSU05_0710	2.4839	phosphotransferase system cellobiose-specific component IIB
SSU05_1489	3.5544	Beta-glucosidase/6-phospho-beta-glucosidase/beta-galactosidase
SSU05_1490	2.9274	phosphotransferase system IIC component, glucose/maltose/N-acetylglucosamine-specific
SSU05_1497	3.1051	3-carboxymuconate cyclase
SSU05_1607	2.016	phosphomannose isomerase
SSU05_2064	2.5809	Type II secretory pathway, pullulanase PulA and related glycosidases

SSU05_2065	2.9013	Type II secretory pathway, pullulanase PulA and related glycosidases
SSU05_1140	2.741	D-beta-hydroxybutyrate permease
SSU05_0706	2.5617	sugar metabolism transcriptional regulator

Cell cycle control, mitosis and meiosis

upregulated

ID	FC	Description
SSU05_0417	2.962	cell division initiation protein
SSU05_0872	3.2971	putative cytoplasmic protein
SSU05_1509	4.2736	septation ring formation regulator EzrA

downregulated

ID	FC	Description
SSU05_0479	2.0153	Actin-like ATPase involved in cell division
SSU05_1091	2.103	transglutaminase/protease-like domain-containing protein

Cell wall/membrane biogenesis

upregulated

ID	FC	Description
SSU05_0019	2.1961	rod shape-determining protein MreC
SSU05_0195	2.0096	phosphosugar isomerase
SSU05_1430	3.3022	large-conductance mechanosensitive channel
SSU05_1720	3.0652	UDP-N-acetylmuramate--L-alanine ligase
SSU05_2173	5.3557	FOG: LysM repeat
SSU05_0719	4.6203	dihydrodipicolinate synthase

downregulated

ID	FC	Description
SSU05_0329	30.5394	hypothetical protein
SSU05_0549	5.5242	glucosamine--fructose-6-phosphate aminotransferase
SSU05_0965	3.3171	agglutinin receptor
SSU05_1170	2.6651	UDP-N-acetylglucosamine 1-carboxyvinyltransferase
SSU05_1706	2.5318	glutamate racemase
SSU05_2100	2.7248	hypothetical protein

Coenzyme transport and metabolism

upregulated

ID	FC	Description
SSU05_0303	2.8179	lipoate-protein ligase A
SSU05_1077	2.6834	dihydrofolate reductase
SSU05_1088	9.7555	pantothenate kinase
SSU05_1752	3.3516	1,4-dihydroxy-2-naphthoate octaprenyltransferase
SSU05_1753	2.3132	thiamine biosynthesis lipoprotein
SSU05_1755	3.4638	geranylgeranyl pyrophosphate synthase
SSU05_1756	3.838	geranylgeranyl pyrophosphate synthase

downregulated

ID	FC	Description
SSU05_0688	12.0333	phosphopantothenoylcysteine decarboxylase

SSU05_0689	19.7877	phosphopantothenate--cysteine ligase
SSU05_0835	3.5825	dihydrofolate reductase
SSU05_1141	2.2441	putative 2-amino-4-hydroxy-6-hydroxymethylpteridine pyrophosphokinase
SSU05_1144	4.0477	GTP cyclohydrolase I
SSU05_1466	3.1073	SAM-dependent methyltransferase
SSU05_1580	4.1754	putative pyridoxal kinase
SSU05_1680	2.5565	phosphopantetheine adenylyltransferase
SSU05_2043	5.823	putative 5-formyltetrahydrofolate cyclo-ligase

Defense mechanisms

upregulated

ID	FC	Description
SSU05_0748	8.4683	ABC-type multidrug transport system, ATPase component
SSU05_0946	2.6404	hypothetical protein
SSU05_0947	2.7863	ABC-type multidrug transport system, ATPase and permease component
SSU05_0960	2.307	SalB
SSU05_1381	2.0343	peptide ABC transporter ATPase
SSU05_1405	2.6893	putative ABC transporter
SSU05_1406	4.6278	ABC-type multidrug transport system, ATPase and permease component
SSU05_1995	2.8648	multidrug ABC transporter, ATP-binding protein

downregulated

ID	FC	Description
SSU05_0694	2.0113	putative HsdM
SSU05_0798	2.5717	putative ABC transporter, ATP-binding protein
SSU05_0816	44.7548	hypothetical protein
SSU05_0817	3.5098	hypothetical protein
SSU05_0818	3.1897	Na+-driven multidrug efflux pump
SSU05_0891	2.0552	peptide ABC transporter ATPase
SSU05_1855	4.0664	ABC-type multidrug transport system, ATPase component

Energy production and conversion

upregulated

ID	FC	Description
SSU05_0518	2.3705	coenzyme F420-dependent N5,N10-methylene tetrahydromethanopterin reductase-like protein
SSU05_0716	5.3054	glycerol dehydrogenase and related enzyme
SSU05_1057	2.3987	phosphotransacetylase
SSU05_1171	3.3042	mitochondrial delta subunit
SSU05_1172	3.7993	F0F1 ATP synthase subunit beta
SSU05_1173	6.4711	F0F1 ATP synthase subunit gamma
SSU05_1174	5.2818	F0F1 ATP synthase subunit alpha
SSU05_1175	2.1429	mitochondrial oligomycin sensitivity protein
SSU05_1757	2.9458	NADH dehydrogenase, FAD-containing subunit

downregulated

ID	FC	Description
SSU05_0135	3.0337	acetate kinase
SSU05_0511	3.3827	hypothetical protein
SSU05_0512	4.9612	glutathione reductase
SSU05_1076	2.4501	L-lactate dehydrogenase
SSU05_1272	2.3985	ferredoxin
SSU05_0140	3.5105	Thiol-disulfide isomerase and thioredoxin

Inorganic ion transport and metabolism

upregulated

ID	FC	Description
SSU05_0217	2.0592	ABC-type nitrate/sulfonate/bicarbonate transport system, ATPase component
SSU05_0309	2.6318	cation transport ATPase
SSU05_0658	2.2082	hypothetical protein
SSU05_0991	6.3813	rhodanese-related sulfurtransferase
SSU05_1447	3.324	arsenate reductase
SSU05_1539	3.159	manganese-dependent superoxide dismutase
SSU05_1689	3.5544	Dpr
SSU05_2083	28.6387	zinc ABC transporter, permease protein
SSU05_2084	14.5822	Mn2+/Zn2+ ABC transporter permease
SSU05_2085	10.7418	unknown pir||T45470

downregulated

ID	FC	Description
SSU05_0111	2.6361	ABC-type Mn2+/Zn2+ transport system, permease component
SSU05_0646	2.4818	ABC-type Fe3+-siderophore transport system, permease component
SSU05_0647	2.9343	ABC-type Fe3+-siderophore transport system, permease component
SSU05_0649	2.2481	ABC-type Fe3+-hydroxamate transport system, periplasmic component
SSU05_1302	2.0697	divalent heavy-metal cations transporter
SSU05_1348	2.9795	cation transport ATPase
SSU05_1389	2.1096	tellurite resistance protein TehB
SSU05_1669	3.7025	ABC-type molybdenum transport system, ATPase component/photorepair protein PhrA
SSU05_1683	2.7524	rhodanese-related sulfurtransferase
SSU05_2032	16.3359	hypothetical protein

Intracellular trafficking and secretion

upregulated

ID	FC	Description
SSU05_1216	2.4068	preprotein translocase subunit YajC
SSU05_1984	3.4087	preprotein translocase subunit SecE

SSU05_1550	2.3942	ATP-dependent Clp protease proteolytic subunit
**downregulated**		
ID	FC	Description
SSU05_0131	2.8956	competence protein ComGF
SSU05_1854	2.8966	ABC transporter permease
SSU05_0987	5.4487	Rossmann fold nucleotide-binding protein involved in DNA uptake
SSU05_0988	11.4588	Rossmann fold nucleotide-binding protein involved in DNA uptake
**Lipid transport and metabolism**		
**upregulated**		
ID	FC	Description
SSU05_0652	2.0528	1-acyl-sn-glycerol-3-phosphate acyltransferase
SSU05_1569	4.033	Acyl carrier protein phosphodiesterase
SSU05_1570	2.2624	Acyl carrier protein phosphodiesterase
SSU05_1156	12.7592	D-mannonate oxidoreductase
SSU05_1671	4.4913	Type II secretory pathway, prepilin signal peptidase PulO and related peptidases
**downregulated**		
ID	FC	Description
SSU05_0239	2.1293	Acyl-coenzyme A synthetases/AMP-(fatty) acid ligase
SSU05_1797	5.8236	Acetyl-CoA carboxylase beta subunit
SSU05_1798	7.4644	Acetyl-CoA carboxylase beta subunit
SSU05_1799	7.2351	acetyl-CoA carboxylase biotin carboxylase subunit
SSU05_1800	5.3883	3-hydroxymyristoyl/3-hydroxydecanoyl-(acyl carrier protein) dehydratase
SSU05_1801	7.8289	acetyl-CoA carboxylase biotin carboxyl carrier protein subunit
SSU05_1804	16.5302	(acyl-carrier-protein) S-malonyltransferase
SSU05_1807	9.0578	3-oxoacyl-(acyl carrier protein) synthase III
SSU05_1809	36.6113	enoyl-CoA hydratase
SSU05_0003	4.9699	sphingosine kinase and enzymes related to diacylglycerol kinase
SSU05_1802	5.9343	3-oxoacyl-(acyl carrier protein) synthase II
SSU05_1806	9.2528	acyl carrier protein
SSU05_1803	12.347	3-ketoacyl-(acyl-carrier-protein) reductase
**Nucleotide transport and metabolism**		
**upregulated**		
ID	FC	Description
SSU05_0027	2.4229	phosphoribosylformylglycinamidine synthase domain-containing protein
SSU05_0028	2.5703	amidophosphoribosyltransferase
SSU05_0030	2.4207	folate-dependent phosphoribosylglycinamide formyltransferase PurN

SSU05_0031	2.0014	phosphoribosyl glycinamide transformylase-N
SSU05_0032	2.3699	bifunctional phosphoribosylaminoimidazolecarboxamide formyltransferase/IMP cyclohydrolase
SSU05_0033	3.6053	phosphoribosylamine--glycine ligase
SSU05_0034	4.044	phosphoribosylcarboxyaminoimidazole (NCAIR) mutase
SSU05_0035	2.8575	phosphoribosylaminoimidazole carboxylase ATPase subunit
SSU05_0039	2.074	adenylosuccinate lyase
SSU05_0271	4.6292	NTP pyrophosphohydrolase including oxidative damage repair enzyme
SSU05_0538	4.3775	adenosine deaminase
SSU05_0729	14.2952	oxygen-sensitive ribonucleoside-triphosphate reductase
SSU05_1000	3.7508	putative 5'-nucleotidase
SSU05_1445	2.0643	purine nucleoside phosphorylase
SSU05_1446	2.6215	purine nucleoside phosphorylase
SSU05_1538	8.6247	putative 5'-nucleotidase
SSU05_2183	8.5071	inosine 5'-monophosphate dehydrogenase
SSU05_0021	3.7477	phosphoribosylpyrophosphate synthetase

downregulated

ID	FC	Description
SSU05_0014	2.3624	hypoxanthine-guanine phosphoribosyltransferase
SSU05_0661	3.8709	thymidylate kinase
SSU05_0789	7.8755	bifunctional pyrimidine regulatory protein PyrR uracil phosphoribosyltransferase
SSU05_0790	4.9662	aspartate carbamoyltransferase catalytic subunit
SSU05_0815	94.9164	guanosine 5'-monophosphate oxidoreductase
SSU05_0834	2.8664	thymidylate synthase
SSU05_1145	3.7946	NTP pyrophosphohydrolase including oxidative damage repair enzymes
SSU05_1207	2.9663	ribonucleotide-diphosphate reductase subunit alpha
SSU05_1327	2.0961	uridylate kinase
SSU05_1935	2.41	xanthine/uracil permease
SSU05_1937	3.2321	hypothetical protein
SSU05_1966	3.4573	adenylosuccinate synthase
SSU05_0791	3.7124	carbamoyl phosphate synthase small subunit
SSU05_1065	3.1158	ribose-phosphate pyrophosphokinase

Posttranslational modification, protein turnover, chaperones

upregulated

ID	FC	Description
SSU05_0299	3.404	molecular chaperone GrpE (heat shock protein)
SSU05_1667	4.2854	pyruvate-formate lyase activating enzyme
SSU05_1875	2.3886	ABC-type transport system involved in Fe-S cluster assembly, permease component
SSU05_1550	2.3942	ATP-dependent Clp protease proteolytic subunit

downregulated

ID	FC	Description
SSU05_0015	2.2278	ATP-dependent Zn protease
SSU05_0147	6.9452	co-chaperonin GroES
SSU05_0148	2.7925	putative chaperonin GroEL
SSU05_0153	6.1349	metalloendopeptidase
SSU05_0505	3.8553	collagenase-like protease
SSU05_0645	2.0869	glutathione peroxidase
SSU05_0705	2.3218	pyruvate-formate lyase-activating enzyme
SSU05_1206	6.3519	NrdH-redoxin
SSU05_2114	2.7847	organic radical activating protein
SSU05_0140	3.5105	Thiol-disulfide isomerase and thioredoxin
SSU05_1671	4.4913	Type II secretory pathway, prepilin signal peptidase PulO and related peptidases

Replication, recombination and repair

upregulated

ID	FC	Description
SSU05_0208	4.7152	hypothetical protein
SSU05_0235	2.4761	mismatch repair ATPase
SSU05_1226	3.1549	transposase
SSU05_2123	4.8102	DNA mismatch repair protein MutS
SSU05_1722	2.141	SNF2 family DNA/RNA helicase
SSU05_0428	2.4717	Serine/threonine protein kinase

downregulated

ID	FC	Description
SSU05_0133	3.6841	adenine-specific DNA methylase
SSU05_0145	2.7432	single-stranded DNA-binding protein
SSU05_0424	2.5193	primosome assembly protein PriA
SSU05_0437	2.7369	superfamily II DNA/RNA helicase
SSU05_0536	2.0444	hypothetical protein
SSU05_0537	3.1364	hypothetical protein
SSU05_0685	2.2271	IS200 family transposase
SSU05_0813	3.4359	EndoIII-related endonuclease
SSU05_1098	4.4309	transposase
SSU05_1198	2.9465	hypothetical protein
SSU05_1345	3.657	transposase
SSU05_1424	2.0074	transposase
SSU05_1507	3.4411	transposase
SSU05_1529	3.7048	prophage Lp3 protein 1, integrase
SSU05_1637	2.7404	IS200 family transposase
SSU05_1645	3.165	transposase
SSU05_1646	2.1532	transposase
SSU05_1681	3.3037	hypothetical protein
SSU05_1835	2.1021	hypothetical protein
SSU05_1863	2.2046	transposase

SSU05_1913	4.2423	transposase
SSU05_1954	4.3633	DNA polymerase III PolC
SSU05_0987	5.4487	Rossmann fold nucleotide-binding protein involved in DNA uptake
SSU05_0988	11.4588	Rossmann fold nucleotide-binding protein involved in DNA uptake
SSU05_0344	2.1505	L-asparaginase/ Glu-tRNAGln amidotransferase subunit D
SSU05_0345	6.747	L-asparaginase/ Glu-tRNAGln amidotransferase subunit D
SSU05_1574	3.4537	superfamily II DNA/RNA helicase
SSU05_1634	2.1715	superfamily II DNA/RNA helicase

Signal transduction mechanisms

upregulated

ID	FC	Description
SSU05_0906	2.2888	NisK
SSU05_0305	2.1106	amino acid ABC transporter periplasmic protein
SSU05_0552	3.6411	amino acid ABC transporter, amino acid-binding protein
SSU05_0907	2.1292	NisR

downregulated

ID	FC	Description
SSU05_0430	4.7379	Signal transduction histidine kinase
SSU05_1017	11.8352	amino acid ABC transporter periplasmic protein
SSU05_1018	43.1707	putative amino acid transporter, amino acid-binding protein
SSU05_2069	49.8037	amino acid ABC transporter periplasmic protein
SSU05_0432	3.3087	response regulator
SSU05_2148	3.7154	response regulator

Secondary metabolites biosynthesis, transport and catabolism

upregulated

ID	FC	Description
SSU05_1156	12.7592	D-mannonate oxidoreductase
SSU05_0351	3.6412	nicotinamidase-like amidase

downregulated

ID	FC	Description
SSU05_0003	4.9699	sphingosine kinase and enzymes related to diacylglycerol kinase
SSU05_1802	5.9343	3-oxoacyl-(acyl carrier protein) synthase II
SSU05_1806	9.2528	acyl carrier protein
SSU05_1803	12.347	3-ketoacyl-(acyl-carrier-protein) reductase
SSU05_1493	2.2565	Alpha-acetolactate decarboxylase

Transcription

upregulated

ID	FC	Description
SSU05_0167	2.1991	transcriptional regulator
SSU05_0187	3.2751	transcriptional antiterminator
SSU05_0298	4.5296	heat-inducible transcription repressor

SSU05_0318	3.2479	transcriptional regulator
SSU05_0323	2.0404	hypothetical protein
SSU05_0528	5.7397	sigma24 homolog
SSU05_0655	2.7418	hypothetical protein
SSU05_0905	2.2033	Cro/CI family transcriptional regulator
SSU05_0966	3.9654	transcriptional regulator
SSU05_1136	2.2984	transcriptional regulator
SSU05_1232	9.3908	Cro/CI family transcriptional regulator
SSU05_1341	2.4344	transcriptional regulator
SSU05_1372	4.021	transcriptional regulator
SSU05_1573	3.3423	transcriptional regulator
SSU05_1745	9.5966	transcriptional regulator
SSU05_1819	3.2021	transcriptional regulator
SSU05_2066	5.9249	transcriptional regulator
SSU05_0822	18.7725	sugar metabolism transcriptional regulator
SSU05_1045	4.194	sugar metabolism transcriptional regulator
SSU05_1259	2.2231	N-acetylglucosamine-6-phosphate deacetylase
SSU05_1722	2.141	SNF2 family DNA/RNA helicase
SSU05_0428	2.4717	Serine/threonine protein kinase
SSU05_0907	2.1292	NisR
**downregulated**		
ID	FC	Description
SSU05_0095	3.1009	DNA-directed RNA polymerase subunit alpha
SSU05_0109	2.0768	transcriptional regulator
SSU05_0401	4.232	transcriptional regulator
SSU05_0411	2.6651	cold shock protein
SSU05_0503	2.0525	putative mercuric resisitant regulatory protein
SSU05_0608	3.2662	transcriptional regulator
SSU05_1491	6.151	transcriptional antiterminator
SSU05_1527	3.821	transcriptional regulator
SSU05_1808	27.2478	transcriptional regulator
SSU05_1848	2.0598	nucleic-acid-binding protein implicated in transcription termination
SSU05_1849	2.4761	transcription elongation factor NusA
SSU05_2039	2.3282	transcriptional regulator
SSU05_0562	2.7625	hypothetical protein
SSU05_0706	2.5617	sugar metabolism transcriptional regulator
SSU05_0432	3.3087	response regulator
SSU05_2148	3.7154	response regulator
SSU05_0344	2.1505	L-asparaginase/ Glu-tRNAGln amidotransferase subunit D
SSU05_0345	6.747	L-asparaginase/ Glu-tRNAGln amidotransferase subunit D
SSU05_1574	3.4537	superfamily II DNA/RNA helicase
SSU05_1634	2.1715	superfamily II DNA/RNA helicase
**Translation**		

upregulated

ID	FC	Description
SSU05_0276	2.6579	50S ribosomal protein L33
SSU05_0277	2.2635	50S ribosomal protein L32
SSU05_0304	2.5809	amidase
SSU05_0340	2.9263	50S ribosomal protein L28
SSU05_0352	2.5874	50S ribosomal protein L19
SSU05_0434	2.6567	hypothetical protein
SSU05_0439	20.2573	ribosome-associated protein Y (PSrp-1)
SSU05_0459	4.2636	valyl-tRNA synthetase
SSU05_0922	2.4244	translation elongation factor (GTPases)
SSU05_1433	3.6533	30S ribosomal protein S21
SSU05_1902	3.1961	tRNA/rRNA methyltransferase
SSU05_1961	2.5178	prolyl-tRNA synthetase
SSU05_2027	2.8713	glutamyl-tRNA synthetase
SSU05_2156	3.2091	30S ribosomal protein S4

downregulated

ID	FC	Description
SSU05_0080	2.025	30S ribosomal protein S17
SSU05_0083	2.3782	50S ribosomal protein L5
SSU05_0088	2.0933	50S ribosomal protein L30
SSU05_0093	5.3218	30S ribosomal protein S13
SSU05_0094	6.0241	30S ribosomal protein S11
SSU05_0096	3.6083	ribosomal protein L17
SSU05_0097	4.1195	ribosomal protein L17
SSU05_0445	2.6331	hypothetical protein
SSU05_0488	2.1555	histone acetyltransferase HPA2-like acetyltransferase
SSU05_0644	3.7278	16S rRNA uridine-516 pseudouridylate synthase family protein
SSU05_0901	2.2863	tRNA (uracil-5-)-methyltransferase Gid
SSU05_0983	2.9559	50S ribosomal protein L7/L12
SSU05_1114	2.7923	pseudouridine synthase
SSU05_1115	3.234	pseudouridine synthase
SSU05_1269	3.1282	50S ribosomal protein L35
SSU05_1270	2.5099	translation initiation factor IF-3
SSU05_1332	2.0422	50S ribosomal protein L11
SSU05_1412	2.3997	peptide chain release factor 2
SSU05_1535	2.151	30S ribosomal protein S14
SSU05_1859	4.451	histone acetyltransferase HPA2-like acetyltransferase
SSU05_1944	2.9691	peptide deformylase
SSU05_2015	4.3645	ribonuclease P
SSU05_0344	2.1505	L-asparaginase/ Glu-tRNAGln amidotransferase subunit D
SSU05_0345	6.747	L-asparaginase/ Glu-tRNAGln amidotransferase subunit D
SSU05_1574	3.4537	superfamily II DNA/RNA helicase

SSU05_1634	2.1715	superfamily II DNA/RNA helicase

General function prediction only

upregulated

ID	FC	Description
SSU05_0257	2.5075	ABC-type uncharacterized transport system, permease component
SSU05_0279	2.0018	alcohol dehydrogenase
SSU05_0321	2.0442	dehydrogenase
SSU05_0457	2.0689	lactoylglutathione lyase and related lyases
SSU05_0625	42.2696	histone acetyltransferase HPA2-like acetyltransferase
SSU05_0720	2.1005	pyridine nucleotide-disulfide family oxidoreductase
SSU05_0727	2.6577	hypothetical protein
SSU05_0902	6.6691	HAD superfamily hydrolase
SSU05_0992	12.6783	NAD(FAD)-dependent dehydrogenase
SSU05_0998	2.4683	Fe-S-cluster oxidoreductase
SSU05_1069	2.6811	redox-sensing transcriptional repressor Rex
SSU05_1079	3.0528	ABC transporter permease
SSU05_1080	3.5211	ABC-type uncharacterized transport system, permease component
SSU05_1081	3.0327	ABC-type uncharacterized transport system, ATPase component
SSU05_1155	9.3708	phosphatase
SSU05_1253	2.8473	ABC-type uncharacterized transport system, ATPase component
SSU05_1255	2.292	ABC-type uncharacterized transport system, permease component
SSU05_1256	2.1843	hypothetical protein
SSU05_1313	4.1438	ribonuclease Z
SSU05_1376	2.5582	CoA-binding protein
SSU05_1397	3.5783	GTP-binding protein Era
SSU05_1467	3.1777	hypothetical protein
SSU05_1901	3.6235	hypothetical protein
SSU05_1932	4.1534	glucokinase regulatory protein
SSU05_1952	4.2215	hypothetical protein
SSU05_1968	5.4364	DNA nuclease
SSU05_2070	10.8151	surface antigen

downregulated

ID	FC	Description
SSU05_0042	2.6014	metal-dependent membrane protease
SSU05_0203	2.749	putative NADH-flavin reductase
SSU05_0204	3.4452	putative NADH-flavin reductase
SSU05_0346	4.334	HAD superfamily hydrolase
SSU05_0438	2.7106	amidophosphoribosyltransferase
SSU05_0443	4.9409	recombination regulator RecX

SSU05_0482	2.0571	TIM-barrel fold family protein
SSU05_0509	74.9257	hypothetical protein
SSU05_0510	12.5312	hypothetical protein
SSU05_0533	2.1893	HD superfamily phosphohydrolase
SSU05_0607	4.1862	flavoprotein
SSU05_0838	2.2225	GTPase
SSU05_1182	3.3516	permease
SSU05_1264	2.1167	SAM-dependent methyltransferase
SSU05_1427	3.1216	metal-sulfur cluster biosynthetic protein
SSU05_1496	2.44	permease
SSU05_1533	2.8545	hypothetical protein
SSU05_1571	2.0009	histone acetyltransferase HPA2-like acetyltransferase
SSU05_1613	3.821	aldo/keto reductase family oxidoreductase
SSU05_1632	3.5045	dehydrogenase and related proteins
SSU05_1644	3.0114	histone acetyltransferase HPA2-like acetyltransferase
SSU05_1710	3.7302	putative integral membrane protein
SSU05_1711	3.2899	hypothetical protein
SSU05_1719	2.4453	histone acetyltransferase HPA2-like acetyltransferase
SSU05_1766	2.5654	Phage envelope protein
SSU05_1805	36.1417	2-nitropropane dioxygenase-like protein
SSU05_1810	6.5695	phosphatase/phosphohexomutase
SSU05_1860	6.1987	ATPase or kinase
SSU05_1861	2.2327	kinase related to dihydroxyacetone kinase
SSU05_2042	9.405	hypothetical protein
SSU05_2115	2.3668	acetyltransferase
SSU05_2127	5.6581	surface antigen
SSU05_2169	3.63	ketosteroid isomerase-like protein
SSU05_2187	2.2784	ABC transporter ATPase

others

upregulated

ID	FC	Description
SSU05_0020	8.5014	hypothetical protein
SSU05_0108	3.3349	hypothetical protein
SSU05_0173	2.4361	hypothetical protein
SSU05_0178	2.2275	Epf-like protein
SSU05_0180	3.0571	hypothetical protein
SSU05_0186	2.6435	hypothetical protein
SSU05_0189	3.9259	ascorbate-specific PTS system enzyme IIC
SSU05_0196	2.8858	hypothetical protein
SSU05_0198	2.8517	methyl-accepting chemotaxis protein
SSU05_0207	4.6631	hypothetical protein
SSU05_0213	5.904	hypothetical protein
SSU05_0229	4.0101	FOG: LysM repeat
SSU05_0242	2.7613	transcriptional regulator PlcR, putative

SSU05_0261	2.2947	hypothetical protein
SSU05_0267	2.9149	putative effector of murein hydrolase
SSU05_0315	3.4097	hypothetical protein
SSU05_0316	2.2706	hypothetical protein
SSU05_0317	2.0404	hypothetical protein
SSU05_0458	2.3487	shikimate kinase
SSU05_0460	2.9311	hypothetical protein
SSU05_0461	2.6912	hypothetical protein
SSU05_0504	3.023	hypothetical protein
SSU05_0529	6.9581	hypothetical protein
SSU05_0553	4.0107	hypothetical protein
SSU05_0586	2.4724	hypothetical protein
SSU05_0588	5.1615	transposase
SSU05_0594	2.1206	ATPase involved in DNA repair
SSU05_0595	2.2061	ATPase involved in DNA repair
SSU05_0628	125.049	hypothetical protein
SSU05_0656	2.8183	FOG: CBS domain
SSU05_0659	3.5932	hypothetical protein
SSU05_0673	10.4945	hypothetical protein
SSU05_0674	2.0521	hypothetical protein
SSU05_0679	2.5182	hypothetical protein
SSU05_0726	2.4271	hypothetical protein
SSU05_0747	8.8862	permease
SSU05_0780	3.7554	branched-chain amino acid permease
SSU05_0845	9.3292	hypothetical protein
SSU05_0945	3.0369	hypothetical protein
SSU05_0951	2.2291	DNA recombinase, putative
SSU05_0967	5.1697	hypothetical protein
SSU05_0971	2.1604	ABC-type cobalt transport system, permease component CbiQ and related transporters
SSU05_1048	2.2425	integrase
SSU05_1116	3.9801	hypothetical protein
SSU05_1120	2.7422	histone acetyltransferase HPA2-like acetyltransferase
SSU05_1129	2.0373	hemolysin III homolog
SSU05_1132	2.0887	hypothetical protein
SSU05_1133	2.716	hypothetical protein
SSU05_1134	2.5186	hypothetical protein
SSU05_1165	2.8734	sugar transporter, putative
SSU05_1199	2.4382	hypothetical protein
SSU05_1215	3.4117	hyaluronidase
SSU05_1220	5.4084	hypothetical protein
SSU05_1227	6.5187	metal-dependent membrane protease
SSU05_1228	6.6548	hypothetical protein
SSU05_1229	7.8255	hypothetical protein

SSU05_1230	5.7338	hypothetical protein
SSU05_1231	7.3876	hypothetical protein
SSU05_1233	8.6226	surface antigen negative regulator Par
SSU05_1236	2.7961	alanyl-tRNA synthetase
SSU05_1311	2.7457	hypothetical protein
SSU05_1351	5.3842	hypothetical protein
SSU05_1377	3.1555	glucose-6-phosphate isomerase
SSU05_1379	2.3278	hypothetical protein
SSU05_1382	12.9326	superfamily I DNA/RNA helicase
SSU05_1403	11.8065	hemolysin
SSU05_1417	5.7478	hypothetical protein
SSU05_1442	6.3454	hypothetical protein
SSU05_1456	3.9388	hypothetical protein
SSU05_1457	2.8783	hypothetical protein
SSU05_1458	3.0067	hypothetical protein
SSU05_1481	4.5014	hypothetical protein
SSU05_1482	3.5156	hypothetical protein
SSU05_1541	14.1412	hypothetical protein
SSU05_1549	2.2778	hypothetical protein
SSU05_1572	4.7293	integral membrane protein
SSU05_1578	2.2257	hypothetical protein
SSU05_1603	2.096	Signal recognition particle GTPase
SSU05_1614	2.7012	hypothetical protein
SSU05_1659	2.8065	hypothetical protein
SSU05_1697	2.0441	rRNA methyltransferase
SSU05_1724	2.3886	hypothetical protein
SSU05_1740	2.4431	ABC-type uncharacterized transport system, ATPase component
SSU05_1746	2.8848	hemolysin-like protein
SSU05_1747	8.7676	hypothetical protein
SSU05_1748	12.8667	hypothetical protein
SSU05_1749	10.046	major facilitator superfamily permease
SSU05_1750	8.0184	hypothetical protein
SSU05_1751	2.5621	hypothetical protein
SSU05_1754	6.2946	major membrane immunogen, membrane-anchored lipoprotein
SSU05_1762	4.9486	hypothetical protein
SSU05_1776	3.7854	permease
SSU05_1872	2.097	hypothetical protein
SSU05_1883	2.8231	hypothetical protein
SSU05_1893	2.9248	hypothetical protein
SSU05_1900	3.0718	hypothetical protein
SSU05_1903	5.8821	hypothetical protein
SSU05_1929	4.4137	hypothetical protein

SSU05_1959	68.7467	hypothetical protein
SSU05_1981	2.0108	hypothetical protein
SSU05_2088	2.5169	hypothetical protein
SSU05_2092	5.2885	hypothetical protein
SSU05_2110	2.5039	hypothetical protein
SSU05_2128	4.6217	major facilitator superfamily permease
SSU05_2190	5.2764	hypothetical protein
SSU05_2191	3.5419	rRNA large subunit methyltransferase
**downregulated**		
ID	FC	Description
SSU05_0040	2.0933	hypothetical protein
SSU05_0069	4.7914	hypothetical protein
SSU05_0079	2.2258	50S ribosomal protein L29
SSU05_0134	2.7647	adenine-specific DNA methylase
SSU05_0139	3.5952	hypothetical protein
SSU05_0143	3.2331	hypothetical protein
SSU05_0146	8.1448	hypothetical protein
SSU05_0154	10.3064	hypothetical protein
SSU05_0158	6.7859	hypothetical protein
SSU05_0159	2.9494	transcriptional regulator
SSU05_0238	11.1368	hypothetical protein
SSU05_0332	2.0979	hypothetical protein
SSU05_0400	3.4862	hypothetical protein
SSU05_0403	4.9664	hypothetical protein
SSU05_0429	5.4538	hypothetical protein
SSU05_0431	4.2308	hypothetical protein
SSU05_0499	3.2559	putative signal peptidase IB
SSU05_0500	5.7178	hypothetical protein
SSU05_0507	64.1716	hypothetical protein
SSU05_0521	2.5831	hypothetical protein
SSU05_0561	9.0359	hypothetical protein
SSU05_0615	2.7664	hypothetical protein
SSU05_0633	4.8578	hypothetical protein
SSU05_0635	2.6826	hypothetical protein
SSU05_0657	6.4588	hypothetical protein
SSU05_0687	12.0688	hypothetical protein
SSU05_0756	5.8797	putative glycerol-3-phosphate acyltransferase PlsY
SSU05_0793	2.0825	SAM-dependent methyltransferase
SSU05_0833	6.4848	hypothetical protein
SSU05_0836	2.2209	hypothetical protein
SSU05_0840	2.2139	hypothetical protein
SSU05_0858	2.6946	hypothetical protein
SSU05_0867	3.9487	hypothetical protein
SSU05_0875	3.2588	putative DNA-binding protein

SSU05_0877	2.4853	hypothetical protein
SSU05_0892	2.3514	ScnG homolog
SSU05_0895	3.3107	hypothetical protein
SSU05_0993	7.1851	hypothetical protein
SSU05_1006	2.0919	hypothetical protein
SSU05_1019	51.1907	hypothetical protein
SSU05_1139	2.4988	hypothetical protein
SSU05_1146	2.2625	hypothetical protein
SSU05_1161	6.3543	transcriptional regulator
SSU05_1183	2.7063	hypothetical protein
SSU05_1208	3.2099	hypothetical protein
SSU05_1209	4.8866	ribonucleotide-diphosphate reductase subunit beta
SSU05_1240	2.1188	hypothetical protein
SSU05_1247	7.1431	hypothetical protein
SSU05_1265	3.7815	phosphoglycerol transferase/alkaline phosphatase superfamily protein
SSU05_1273	2.1287	hypothetical protein
SSU05_1274	2.603	hypothetical protein
SSU05_1306	2.0199	homoserine O-succinyltransferase
SSU05_1420	3.0801	hypothetical protein
SSU05_1434	2.3933	hypothetical protein
SSU05_1455	2.5495	hypothetical protein
SSU05_1464	2.3717	hypothetical protein
SSU05_1465	2.1509	hypothetical protein
SSU05_1488	2.1775	hypothetical protein
SSU05_1506	2.6536	phosphatase
SSU05_1528	3.3052	Gp21 protein
SSU05_1597	2.6592	hypothetical protein
SSU05_1610	2.4019	hypothetical protein
SSU05_1626	2.0465	ABC-type multidrug transport system, ATPase and permease component
SSU05_1633	2.0923	dehydrogenase and related proteins
SSU05_1648	2.2549	hypothetical protein
SSU05_1650	2.2921	hypothetical protein
SSU05_1684	3.5905	hypothetical protein
SSU05_1695	2.9104	hypothetical protein
SSU05_1696	2.3016	hypothetical protein
SSU05_1767	2.371	hypothetical protein
SSU05_1773	49.1598	glutamine amidotransferase, class I
SSU05_1789	2.6654	hypothetical protein
SSU05_1792	6.7985	hypothetical protein
SSU05_1793	10.4233	hypothetical protein
SSU05_1794	22.2153	histone acetyltransferase HPA2-like acetyltransferase
SSU05_1795	35.886	histone acetyltransferase HPA2-like acetyltransferase

SSU05_1796	7.8956	acetyl-CoA carboxylase subunit alpha
SSU05_1826	5.3517	Xaa-Pro aminopeptidase
SSU05_1827	9.573	hypothetical protein
SSU05_1853	3.4627	hypothetical protein
SSU05_1862	5.8691	hypothetical protein
SSU05_1904	3.4017	hypothetical protein
SSU05_1936	2.8992	hypothetical protein
SSU05_1953	4.6998	hypothetical protein
SSU05_1977	3.5276	putative bacterocin transport accessory protein, Bta
SSU05_1978	2.2384	hypothetical protein
SSU05_2052	2.6516	hypothetical protein
SSU05_2189	3.0694	hypothetical protein

Table S5. Strains and plasmids used in this study

Strains or plasmids	Characteristics	Reference or source
Strains		
SC-19	Virulent Chinese *S. suis* serotype 2 isolate, wild-type	This work
Δ*relA*	Gene *relA* inactive	This work
Δ*relQ*	Gene *relQ* inactive	This work
Δ*relA*Δ*relQ*	Gene *relA* and *relQ* inactive	This work
DH5α	Genetically modified *E. coli*, cloning host	In this lab
BL21(DE3)	Genetically modified *E. coli*, expression host	In this lab
Plasmids		
pSET4s	Thermosensitive allelic replacement vector	In this lab
pET28a	His tag fusion expression vector	Novagen
pSET4s::*relA*	A mosaic plasmid designed to inactivate *relA*	This work
pSET4s::*relQ*	A mosaic plasmid designed to inactivate *relQ*	This work
pET28a::*relA*	Recombinant expression plasmid to produce His_6-fused RelA protein	This work
pET28a::*relQ*	Recombinant expression plasmid to produce His_6-fused RelQ protein	This work
pAT18	A plasmid containing an *erm*	In this lab

Table S6. Composition of CDM used in this study

	Component	Concn (mg/liter)		Component	Concn (mg/liter)
1	$FeSO_4 \cdot 7H_2O$	5	3	p-Aminobenzoic acid	0.2
	$Fe(NO_3)_2 \cdot 9H_2O$	1		Biotin	0.2
	K_2HPO_4	200		Folic acid	0.8
	KH_2PO_4	1,000		Niacinamide	1
	$MgSO_4 \cdot 7H_2O$	700		β-Nicotinamide adenine dinucleotide	2.5
	$MnSO_4$	5			

2	DL-Alanine	100		Pantothenate calcium salt	2
	L-Arginine	100		Pyridoxal	1
	L-Aspartic acid	100		Pyridoxamine dihydrochloride	1
	L-Cystine	50		Riboflavin	2
	L-Glutamic acid	100		Thiamine hydrochloride	1
	L-Glutamine	200		Vitamin B_{12}	0.1
	Glycine	100	4	Glucose	10,000
	L-Histidine	100	5	Adenine	20
	L-Isoleucine	100		Guanine hydrochloride	20
	L-Leucine	100		Uracil	20
	L-Lysine	100	6	$CaCl_2 \cdot 6H_2O$	10
	L-Methionine	100		$NaC_2H_3O_2 \cdot 3H_2O$	4500
	L-Phenylalanine	100		L-Cysteine	500
	L-Proline	100		$NaHCO_3$	2500
	Hydroxy-L-proline	100		$NaH_2PO_4 \cdot H_2O$	3195
	L-Serine	100		Na_2HPO_4	7350
	L-Threonine	200			
	L-Tryptophan	100			
	L-Tyrosine	100			
	L-Valine	100			

Table S7. Primers used in this study

Primers	Sequence (5’-3’)	Restriction site	target
General PCR amplification			
relL01:	TAGTAAGCTTTTGGCAATCCGTATCAGC	*Hind* III	Left arm of *relA*
relL02	TCACCTGCAGCGCTGGATTAACAACCTG	*Pst* I	
relR01	TCACCTGCAGCAAGTTCAGAGTCGGTTG	*Pst* I	Right arm of *relA*
relR02	AGGTGAATTCATCCCAGAGTCTCTAAGG	*Eco*R I	
ermF	TCACCTGCAGGAGTGTGTTGATAGTGCA	*Pst* I	*erm*
ermR	AGGTCTGCAGCTTGGAAGCTGTCAGTAG	*Pst* I	
relAF	ATGCGGCCGCAATGAAAGACATAAACTACACTG	*Not I*	ORF of *relA*
relAR	CGCTCGAGTTATCCGTTGGTCCTTTTCA	*Xho* I	
relQF	CGGAATTCGCATGGCAGTATTTGAAAAAGTAC	*Eco*R I	ORF of *relQ*
relQR	CGCTCGAGTTATTTTGTTTTTTCTTCAACATA	*Xho* I	
Real-time RT-PCR			
0155F	CGCGAGCCAGGAAACATC		*gapA*
0155R	CTTTAGAAGCAAAGAAACCTGTAGCTT		
0157F	TGCTTTCATCGCAGGTGCTA		*pgk*
0157R	GAACTTGTCCATCTTCCAAAGCAT		
0336F	AGCTCGTGACAACGGTTATGC		*fbaA*
0336R	CTTAGCTGCACCCATAGAAGTTTG		
0543F	AAGGAATGGAAGTTTATGGAATCAA		*pfkA*

0543R	CGTGCAGACAATTCATGGATATC	
0627F	AGACAGTTTGGTAGCCATGACAGA	*arcC*
0627R	GCCTTCCTTGAGCAATTCATTT	
1064F	GATGCAGGGCAAAATTCTTT	*SSU05_1064*
1064R	AGCAGTTCGTGCGACCTTAC	
1372F	AGGCTACGCTCTAGCAGAACGT	*ccpA*
1372R	CAGCAATCTCATCTTCTGCAACATA	
1802F	CGGCGGGTGCAGTTGA	*fabF*
1802R	CCAGCTGTCTTTGGTGCATAAG	
1807F	GGCGGTCGAGGTGCTAGTC	*fabH*
1807R	GCTGACAGCTTTGTGTCGAGAA	
2054F	CACAAGACGCAGCTTACAAGGA	*tktA*
2054R	CCCATTTCAATTGCCAAACG	
0155F	AGAAGTAAACGCTGCTAT	*gapdh*
0155R	CAAACAATGAACCGAAT	
